# Social determinants of recovery from ongoing symptoms following COVID-19 in two UK longitudinal studies: a prospective cohort study

**DOI:** 10.1136/bmjph-2024-001166

**Published:** 2025-03-20

**Authors:** Nathan J Cheetham, Vicky Bowyer, María Paz García, Ruth C E Bowyer, J D Carpentieri, Andy Guise, Ellen J Thompson, Carole H Sudre, Erika Molteni, Michela Antonelli, Rose S Penfold, Nicholas R Harvey, Liane S Canas, Khaled Rjoob, Benjamin Murray, Eric Kerfoot, Alexander Hammers, Sebastien Ourselin, Emma L Duncan, Claire J Steves, The COVID Symptom Study Biobank Consortium, Michela Antonelli

**Affiliations:** 1 Department Of Twin Research & Genetic Epidemiology, King’s College London, London, UK; 2 The Alan Turing Institute, London, UK; 3 Institute of Education, University College London, London, UK; 4 Department of Population Health Sciences, King’s College London, London, UK; 5 School of Psychology, University of Sussex, Brighton, UK; 6 MRC Unit for Lifelong Health and Ageing, Department of Population Science and Experimental Medicine, University College London, London, UK; 7 Centre for Medical Image Computing, Department of Computer Science, University College London, London, UK; 8 School of Biomedical Engineering & Imaging Sciences, King’s College London, London, UK; 9 Edinburgh Delirium Research Group, Ageing and Health, Usher Institute, The University of Edinburgh, Edinburgh, UK; 10 King's College London & Guy's and St Thomas’ PET Centre, King’s College London, London, UK; 11 Guy's and St Thomas’ NHS Foundation Trust, London, UK

**Keywords:** COVID-19, SARS-CoV-2, Sociodemographic Factors, Social Medicine

## Abstract

**Introduction:**

Social gradients in COVID-19 exposure and severity have been observed internationally. Whether combinations of pre-existing social factors, particularly those that confer cumulative advantage and disadvantage, affect recovery from ongoing symptoms following COVID-19 and long COVID is less well understood.

**Methods:**

We analysed data on self-perceived recovery following self-reported COVID-19 illness in two UK community-based cohorts, COVID Symptom Study Biobank (CSSB) (N=2548) and TwinsUK (N=1334). Causal effects of sociodemographic variables reflecting status prior to the COVID-19 pandemic on recovery were estimated with multivariable Poisson regression models, weighted for inverse probability of questionnaire participation and COVID-19 infection and adjusted for potential confounders. Associations between recovery and social strata comprising combinations of sex, education level and local area deprivation were estimated using the intersectional multilevel analysis of individual heterogeneity and discriminatory accuracy (MAIHDA) approach. Further analyses estimated associations with variables reflecting experiences during the pandemic.

**Results:**

Gradients in recovery from COVID-19 along the lines of social advantage were observed in intersectional MAIHDA models, with predicted probability of recovery lowest in female strata with lowest education and highest deprivation levels (CSSB: 55.1% (95% CI 44.0% to 65.1%); TwinsUK: 73.9% (95% CI 61.1% to 83.0%)) and highest in male strata with highest education and lowest deprivation levels (CSSB: 79.1% (95% CI 71.8% to 85.1%); TwinsUK: 89.7% (95% CI 82.5% to 94.1%)). Associations were not explained by differences in prepandemic health. Adverse employment, financial, healthcare access and personal experiences during the pandemic were also negatively associated with recovery.

**Conclusions:**

Inequalities in likelihood of recovery from COVID-19 were observed, with ongoing symptoms several months after coronavirus infection more likely for individuals with greater social disadvantage prior to the pandemic.

**WHAT IS ALREADY KNOWN ON THIS TOPIC**

**WHAT THIS STUDY ADDS**

- This is the first study to test associations between multiple sociodemographics in combination and recovery, finding increased recovery rates for groups with greater social advantage at the beginning of the COVID-19 pandemic in two distinct UK-based cohorts. Associations are also found between recovery and a wide range of adverse employment, financial, housing, health and social care access, and bereavement experiences during the pandemic, including a dose–response relationship with the number of adverse experiences.

**HOW THIS STUDY MIGHT AFFECT RESEARCH, PRACTICE OR POLICY**

- The study has implications for understanding how individuals’ status within social hierarchies contributes to inequalities in COVID-19 and other illnesses, emphasising the need for multidimensional policy and approaches to addressing social determinants of health.

## Introduction

 Following infection with SARS-CoV-2, some individuals report persistent symptoms for months or years.^1^ Such individuals may self-identify under the collective patient-advocated term ‘long COVID’^2^ and/or meet one of the various clinical definitions created to describe persistent symptoms.^3^,^5^ The rate of full recovery from ongoing post-COVID-19 symptoms has been reported to be low among individuals with severe acute infection and/or long-term symptoms, with estimates varying between 15% and 50% at up to 12 months from infection.^6^,^10^ Nationally representative estimates from the UK Coronavirus Infection Survey estimated 1.9 million individuals (2.8% of UK population) as having self-reported long COVID as of March 2023, and 1.0 million (1.4%) and 361 000 (0.5%) reported the impact of ongoing symptoms on their current daily activities as ‘a little’ or ‘a lot’, respectively.^11^ To date, ‘recovery’ following COVID-19 has generally been defined as the absence of ongoing symptoms related to COVID-19 and has been assessed through self-report survey or inferred from self-report and/or clinical assessment of ongoing symptoms.

While previous studies have investigated associations with recovery rate, most have primarily focused on COVID-19 symptoms and pre-existing health, with few studies examining the effects of sociodemographic characteristics as exposures. The few studies that have looked at this found lower likelihood of recovery for those with lower educational qualification levels,^12^ living in higher deprivation areas^7^ and for female sex,^78 10 13^,^15^ and conflicting trends with age^7 8 13 14^ and race/ethnicity,^7 8^ typically at up to 12 months follow-up. Further studies in Germany and the UK assessing ongoing post-COVID symptoms or illness severity rather than self-perceived recovery directly have also found protective effects against ongoing symptoms for those with higher educational qualification levels,^16^ higher income,^17^ and those employed and economically active.^11^ A UK qualitative study also identified a slow recovery process and socioeconomic challenges to recovery as themes for individuals living with long COVID.^18^

**Figure 1:**
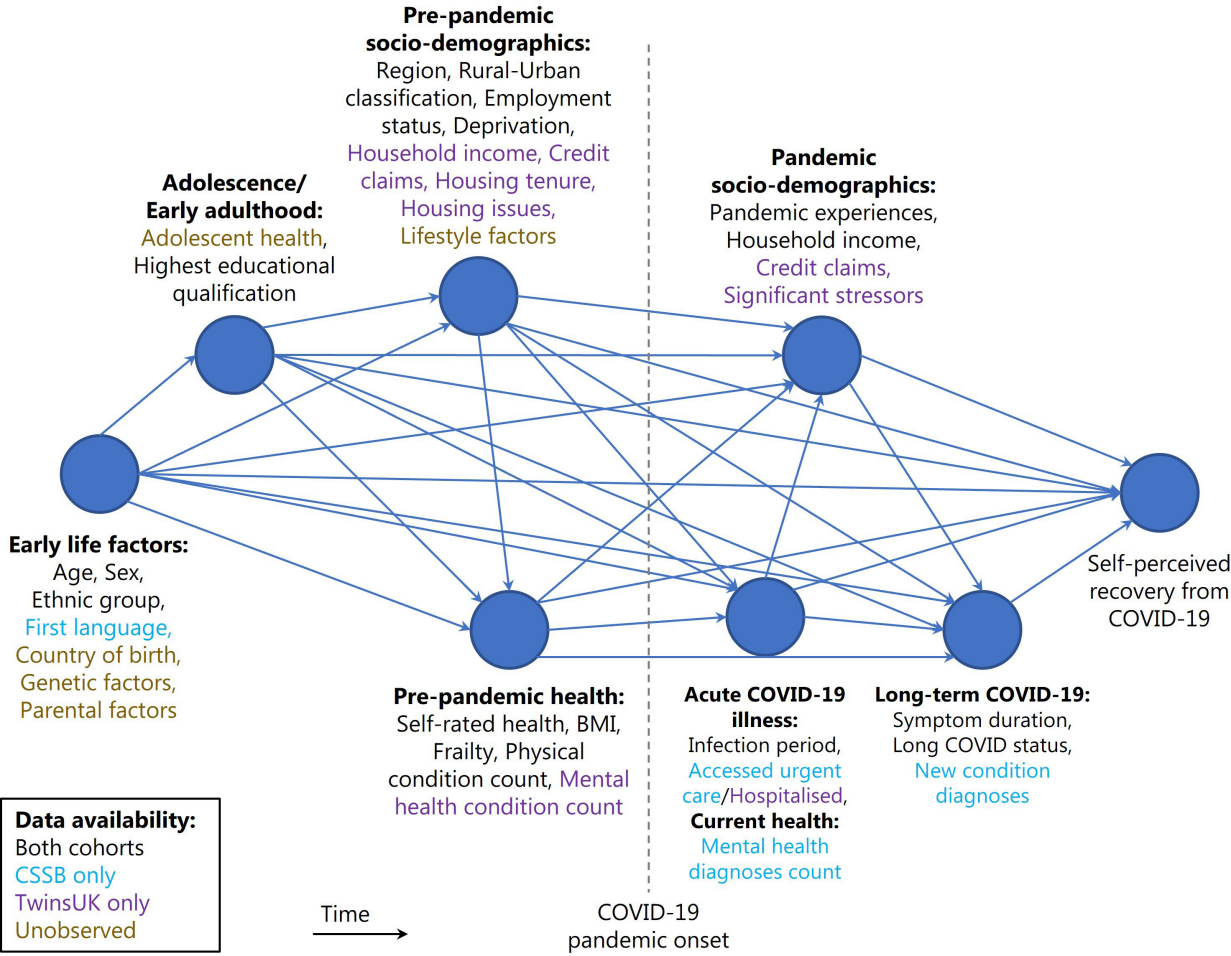
Directed acyclic graph describing hypothesised causal pathways. Proposed directed acyclic graph (DAG) used to generate minimal adjustment variable sets for models estimating the total causal effect of exposure variables on the outcome of self-perceived COVID-19 recovery. Data only available in CSSB or TwinsUK are coloured in blue and purple, respectively, while key unobserved potential confounders are coloured in gold. The DAG is structured approximately in order of data generation/crystallisation from left to right, and variables with similar time of data generation/crystallisation are grouped for clarity into ‘super nodes’. The proposed DAG is ‘saturated’, in that each variable is hypothesised to be caused by all earlier variables. BMI, body mass index; CSSB, COVID Symptom Study Biobank.

**Figure 2:**
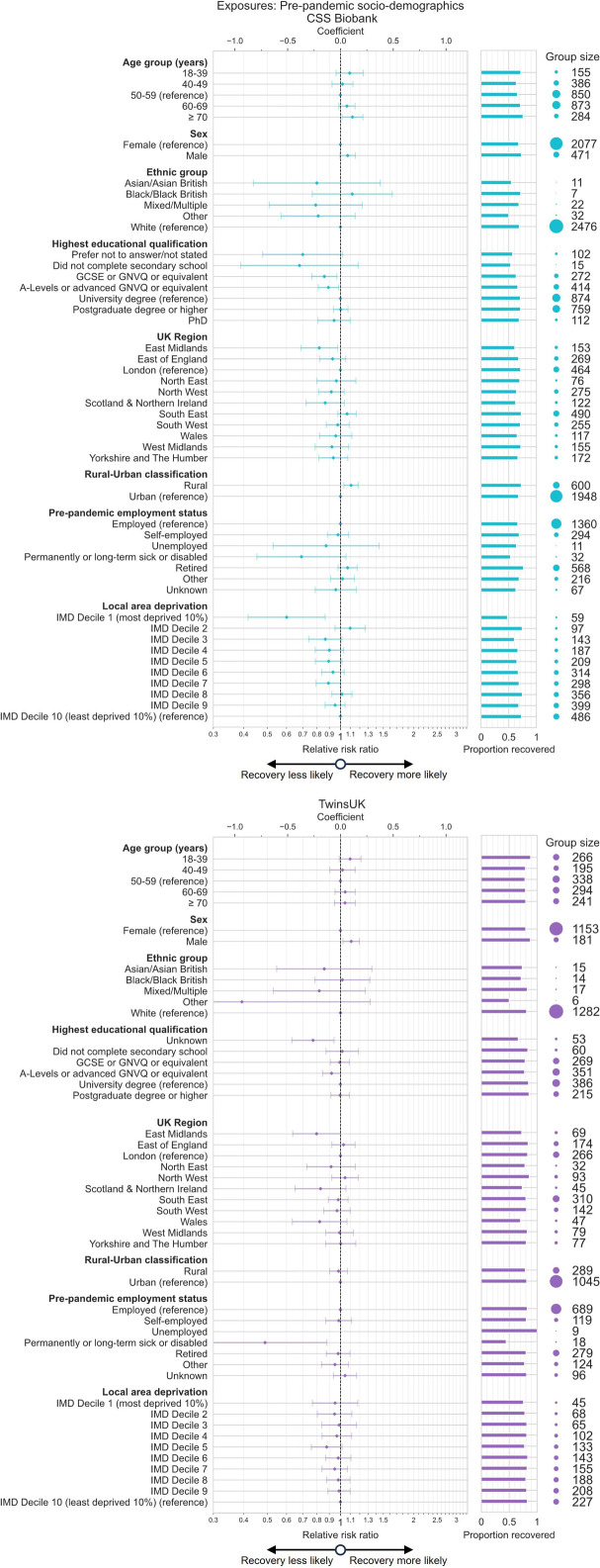
Association between prepandemic sociodemographics and recovery from COVID-19 in CSS Biobank and TwinsUK cohorts. Relative risk ratios (RRs) and 95% CIs from Poisson regression models testing association between recovery from COVID-19 and various prepandemic sociodemographic exposure variables, among individuals with self-reported COVID-19 infection. Unadjusted recovery rates and group sizes from unweighted samples are also shown. Results for each exposure variable originate from models with distinct adjustment variable sets as follows: age: (sex); sex: (age); ethnic group: (age, sex); education: (age, sex, ethnic group, first language (CSSB only)); region: (age, sex, ethnic group, first language (CSSB only), education); rural–urban classification (RUC): (age, sex, ethnic group, first language (CSSB only), education, region); prepandemic employment status: (age, sex, ethnic group, first language (CSSB only), education, region, RUC); local area deprivation: (age, sex, ethnic group, first language (CSSB only), education, region, RUC, prepandemic employment status). Models included participant weighting for inverse probability of questionnaire response and selection into the analysis sample. CSSB, COVID Symptom Study Biobank; IMD, Index of Multiple Deprivation; GCSE, General Certificate of Secondary Education; GNVQ, General National Vocational Qualification.

Given the known importance of such social determinants of health in other chronic conditions such as type 2 diabetes, chronic obstructive pulmonary disease and cardiovascular disease,^19^,^21^ and the implications of ongoing COVID-19 symptoms on daily functioning,^11^ quality of life,^8 22^ cognitive impairment^23^ and increased health risk,^1 24^ as well as socioeconomic consequences such as ability to work,^25 26^ it is important to test whether relationships exist between recovery from COVID-19 and sociodemographic factors. In this study, our objective was to examine whether self-perceived recovery from COVID-19 was associated with: (1) individual sociodemographic factors (illustrated in our directed acyclic graph in figure 1) and (2) combinations of sociodemographic factors using an intersectional approach, within the context of systems of social power and oppression.^27^ Our study is motivated by previous work on the role of cumulative advantage/disadvantage in health inequalities,^28 29^ in the context of the intersectionality framework.^30 31^ We hypothesised that recovery from COVID-19 is associated with exposure to multiple sociodemographic advantages and disadvantages, with individuals exposed to more forms of advantage more likely to recover and those exposed to more forms of disadvantage less likely to recover.

## Methods

### Data sources

Study participants were volunteers from the COVID Symptom Study Biobank (CSSB) and TwinsUK cohorts. Data collection timelines for both cohorts are visualised in online supplemental figure 1.

#### CSSB cohort

CSSB participants were recruited via the COVID Symptom Study app from ZOE (CSS, later renamed ZOE Health Study) launched in the UK on 24 March 2020. All data were collected with informed consent obtained online. Via the CSS app, participants self-report demographic information, symptoms potentially suggestive of COVID-19 infection, any SARS-CoV-2 testing and results, and any vaccinations. CSS participants from across the UK were invited to join the CSSB by email in October to November 2020 and May to June 2021.

CSSB invitation targeted five groups with different statuses at the time of invitation as follows: asymptomatic COVID (positive SARS-CoV-2 test and no associated symptoms); ‘short COVID’ (positive SARS-CoV-2 test and 1–13 days of symptoms); ‘long COVID’ (positive SARS-CoV-2 test and ≥28 days’ symptoms); ‘long non-COVID’ (negative SARS-CoV-2 test and ≥28 days’ symptoms) and ‘healthy non-COVID’ (negative SARS-CoV-2 test and ≤3 days with ≤3 symptoms). Before the invitation, individuals were matched based on minimum Euclidean distance for age, sex and body mass index (BMI) across groups. Due to this targeted approach designed to give five equally sized, matched groups, cohort composition is not representative of population prevalence of COVID-19 and long COVID. Further background details of the cohort are reported elsewhere.^23 32^

CSSB participants were invited (N=8324) to participate in the ‘COVID Reflections—Two Years On’ online questionnaire in August 2022. Questionnaire data were supplemented with data collected at the time of registration with the CSS app, at consent to CSSB, from an earlier CSS app-based questionnaire on mental health in February 2021, and in an earlier CSSB online questionnaire (‘Effects of the Coronavirus Disease (COVID-19) pandemic on life in the UK’) in May 2021. Variables described below were collected as part of the August 2022 questionnaire unless otherwise stated. The CSSB Volunteer Advisory Panel was consulted on the delivery of the August 2022 questionnaire and gave recommendations on invitation and reminder strategies.

#### TwinsUK cohort

TwinsUK is a UK-based national registry of monozygotic and dizygotic twins, with over 15 000 twins registered since 1992.^33^ During the COVID-19 pandemic, TwinsUK participants were invited (N=8869) to a series of ‘COVID-19 personal experience’ (CoPE) questionnaires.^34^ Responses for each round of the CoPE series were collected as follows: (#1) April–May 2020 (#2) July–August 2020, (#3) October–December 2020, (#4) April–July 2021, (#5) November 2021–February 2022 and (#6) April–May 2022. CSSB questionnaires were developed in part from CoPE questionnaires, leading to a high degree of overlap in the types of data collected. COVID-19 questionnaire data were supplemented with data collected as part of TwinsUK routine longitudinal questionnaires both before and during the COVID-19 pandemic.

#### Outcome: self-perceived recovery

Self-perceived recovery following COVID-19 was measured with a single question in CSSB questionnaires and TwinsUK CoPE rounds #3, #4, #5 and #6: “Thinking about the last or only episode of COVID-19 you have had, have you now recovered and are back to normal?”, with the following response options: “Yes, I am back to normal”, “No, I still have some or all my symptoms”. All participants with self-reported COVID-19 were asked about their COVID-19 recovery, except in questionnaires that asked whether an infection was asymptomatic (CSSB August 2022 and CoPE #3, #5), where asymptomatic individuals were not asked about recovery. CSSB analyses considered recovery status at the August 2022 questionnaire only, while TwinsUK analyses took the latest available recovery status from CoPE #3 to #6 for each individual to maximise sample size. Questions relating to long COVID, including self-perceived recovery, were refined from feedback received by the TwinsUK Volunteer Advisory Panel and the Public Involvement Advisory Group of the ‘CONVALESCENCE’ study of long COVID.^35^

#### Sociodemographic, health and COVID-19 illness characteristics

For CSSB participants, sociodemographic characteristics were measured or derived from self-report at registration to the CSS app, CSSB consent or in the August 2022 CSSB questionnaire. Health characteristics were measured or derived from self-report at registration to the CSS app, CSSB consent, in a February 2021 CSS questionnaire, or in the May 2021 or August 2022 CSSB questionnaires. COVID-19 illness characteristics were measured or derived from self-report in the August 2022 and May 2021 CSSB questionnaires.

For TwinsUK participants, sociodemographic and health characteristics were measured or derived from self-report in COVID-19 or routine longitudinal questionnaires. COVID-19 illness characteristics were measured or derived from self-report in COVID-19 questionnaires.

Full details of data sources, question wording and processing prior to analysis are given in online supplemental information sections 1 and 2.

### Eligibility criteria

CSSB and TwinsUK analyses included all individuals with self-reported COVID-19. For all analyses, inclusion criteria were complete data on age, sex, ethnic group, area of residence and completion of the COVID-19 recovery question (in the CSSB August 2022 questionnaire or one or more of the TwinsUK CoPE #3 to #6 questionnaires) as the outcome of interest. Individuals who reported an asymptomatic SARS-CoV-2 infection, or whose longest symptom duration in a COVID-19 episode started less than 84 days before questionnaire completion, were excluded. A full sample selection flow diagram detailing exclusions is given in online supplemental figure 2.

### Statistical analysis

#### Regression models and proposed causal pathways

We used multivariable Poisson regression models with robust errors^36^ to obtain estimates of the effects of individual sociodemographic variables on the outcome of recovery following COVID-19, that is, reporting “Yes, I am back to normal”.

Separate models were run for each exposure variable, including potential confounding variables as appropriate based on the hypothesised directed acyclic graph (DAG) (abridged version in figure 1, full version in online supplemental figure 3), developed using DAGitty software: http://www.dagitty.net/dags.html (full DAGitty code is available openly on GitHub at https://github.com/nathan-cheetham/CSSBiobank_COVIDRecovery).^37^ Models used the ‘HC3’ estimator of coefficient standard errors to account for heteroskedasticity.^38^

Association between recovery and social strata comprising combinations of sex, education level and local area deprivation was estimated using the intersectional multilevel analysis of individual heterogeneity and discriminatory accuracy (MAIHDA) approach.^39 40^ Sex was used as a proxy for gender identity based on data availability within both cohorts. The approach uses a mixed-effects logistic regression model, where a ‘social strata’ variable detailing the explicit combination of sex, education and deprivation acts as a level 2, random (intercept) effects variable, and the individual variables plus potential confounders are included as level 1 fixed effects. This approach was chosen following reports comparing quantitative intersectional methods, finding MAIHDA to be the preferred method for accurate, unbiased estimation of intersections with small sample sizes.^41^ The method assumes that covariates are independent of the social stratum random effect, and that both social stratum random effects and the deviation in outcome for individuals from the mean of their social stratum are normally distributed with a mean of 0.^39 40^ For each social stratum, we present the stratum-level average predicted probability of the outcome generated from the fitted model, which captures both additive and interaction effects. Predicted probabilities are intended to accurately describe the average likelihood of the outcome for particular social intersections, and thereby help to identify intersectional inequalities, rather than identify causal effects. Meanwhile, precision weighting within the modelling approach is intended to protect against extreme values within unadjusted data from social strata with small sample sizes.^40^ The R script used to fit MAIHDA models was adapted from a MAIHDA tutorial.^39 42^ 95% confidence intervals (CIs) for predicted probabilities are approximate only because the model assumes no sampling covariability between the regression coefficients and stratum random effects.

Primary analyses estimated (1) the total causal effects of individual prepandemic sociodemographic variables on the COVID-19 recovery outcome; (2) the association between recovery and social strata comprising combinations of sex, education level and local area deprivation using the intersectional MAIHDA modelling approach and (3) the association between recovery and sociodemographic factors during the pandemic. Sensitivity analyses repeated analyses (1) and (2) with the addition of prepandemic health factors as potential confounders. Secondary analyses estimated the total causal effects of prepandemic health characteristics and COVID-19 illness characteristics on COVID-19 recovery. In all Poisson regression models, potential confounding variables were included based on the hypothesised DAG. P values adjusted for multiple testing are provided in online supplemental information.

#### Generation of inverse probability weights

Models estimating associations with COVID-19 recovery included weights representing the inverse probability of questionnaire response and selection into the analysis sample. Weights were used to reduce potential selection bias from differential response rates and collider bias from conditioning the analysis sample on COVID-19 infection—since infection is a mediating factor between our exposures (prepandemic sociodemographics) and outcome (COVID-19 recovery), any unmeasured factor associated with both COVID-19 infection and our outcome may bias the estimated associations between exposures and outcome.^43 44^ Following methods used in previous CSSB studies,^23^ variable sets that optimised prediction were found using forward sequential feature selection. Individuals with missing data were assigned to a ‘missing data’ category for the given predictor variable that was included in prediction models. Two sets of weights were generated from multivariable logistic regression models, the first optimised prediction of questionnaire response (area under the receiver operating characteristic curve (AUC-ROC) scores of 0.82 for CSSB and 0.88 for TwinsUK models), and the second selection into the analysis sample (AUC-ROC scores of 0.64 for CSSB and 0.67 for TwinsUK models). The two sets of weights were multiplied together and winsorised to limit the influence of individuals with extreme weight values (bottom and top 5% values set equal to 5th and 95th percentile values) prior to use in Poisson regression models.

The CSSB model to predict the probability of questionnaire response comprised the following variables: age group, ethnic group, CSSB invitation round and recruitment group, PRISMA-7 (from the program on research for integrating services for the maintenance of autonomy, PRISMA) scale score (at registration with CSS app),^45^ local area deprivation, number of mental health conditions (from February 2021 CSS questionnaire), and number of non-responses to prior CSSB studies. The CSSB model to predict the probability of selection into the analysis sample comprised: sex, first language, highest educational qualification, region, healthcare professional status, prepandemic employment status, prepandemic self-reported health, PRISMA-7 scale score.

The TwinsUK model to predict the probability of participation in one or more of the CoPE #3 to #6 questionnaires comprised: age group, sex, ethnic group, highest educational qualification, number of non-responses to prior questionnaires and latest available prepandemic data for: region, local area deprivation, household income, BMI, PRISMA-7 score, the number of physical health conditions, the number of mental health conditions. The TwinsUK model to predict the probability of selection into the analysis sample comprised: age group, prepandemic employment status, prepandemic region, prepandemic history of credit/benefit claim, prepandemic health and latest available prepandemic data for PRISMA-7 score and number of mental health conditions.

#### Software

Poisson regression models were fit and figures were created using Python V.3.8.8 and packages: numpy V.1.20.1, pandas V.1.2.4, statsmodels V.0.12.2, scipy V.1.6.2, scikit-learn V.0.24.1, matplotlib V.3.3.4, seaborn V.0.11.1. Intersectional MAIHDA regression models were fit using R V.4.3.0 and packages: haven V.2.5.4, tidyverse V.2.0.0, ggeffects V.1.5.2, lme4 V.1.1.35.2, merTools v0.6.2, labelled V.2.13.0, sjPlot V.2.8.16, Metrics v0.1.4, dplyr V.1.1.4.

### Role of the funding source

The funders of the study had no role in the design of the study, data collection, data analysis, interpretation or writing of the report. All authors had full access to all data within the study. The corresponding authors had final responsibility for the decision to submit for publication.

### Patient and public involvement

Members from the TwinsUK Volunteer Advisory Panel and the Public Involvement Advisory Group of the ‘CONVALESCENCE’ study of long COVID provided feedback and refinement at the design stage of our key outcome question regarding self-perceived recovery from COVID-19. Preliminary results of analyses presented here were also discussed with the CSSB Volunteer Advisory Panel, where feedback informed our reporting and dissemination plans.

## Results

### Sample characteristics

Data from 2548 CSSB participants (of 3731 questionnaire respondents) and 1334 TwinsUK participants (of 5466 questionnaire respondents) with one or more self-reported COVID-19 illnesses were analysed (sample selection shown in online supplemental figure 2).

Across both cohorts, the median age group was 50–59 years old, most individuals were female sex, identified as white ethnic groups, lived in less deprived areas and were employed immediately before the COVID-19 pandemic (table 1). Educational qualification levels among CSSB participants were high in comparison to TwinsUK and the general UK population. General health was self-rated prior to the COVID-19 pandemic as ‘excellent’ or ‘very good’ by just over half in both cohorts (CSSB: 56%, TwinsUK: 58%, from extended sample characteristics, online supplemental tables 1,2).

**Table 1:** Sample characteristics

Variable	CSSB		TwinsUK	
	Group size, N (%)	COVID-19 recovery (%)	Group size, N (%)	COVID-19 recovery (%)
**Total**	2548		1334	
COVID-19 recovery status				
Recovered	1748 (68.6)	68.6	1078 (80.8)	80.8
Not recovered	800 (31.4)		256 (19.2)	
Age (years)				
Median (IQR)	58 (51–65)		56 (44–66)	
Age group (years)				
18–39	155 (6.1)	71.6	266 (19.9)	88.3
40–49	386 (15.1)	63.5	195 (14.6)	79.0
50–59 (reference)	850 (33.4)	65.4	338 (25.3)	78.1
60–69	873 (34.3)	71.1	294 (22.0)	79.3
≥70	284 (11.1)	75.7	241 (18.1)	79.7
Sex				
Female (reference)	2077 (81.5)	67.6	1153 (86.4)	79.7
Male	471 (18.5)	72.8	181 (13.6)	87.8
Ethnic group				
Asian/Asian British	11 (0.4)	54.5	15 (1.1)	73.3
Black/Black British	7 (0.3)	71.4	14 (1.0)	71.4
Mixed/multiple	22 (0.9)	68.2	17 (1.3)	82.4
Other	32 (1.3)	50.0	6 (0.4)	50.0
White groups (reference)	2476 (97.2)	68.9	1282 (96.1)	81.1
Highest educational qualification				
Prefer not to answer/not stated	102 (4.0)	56.9	53 (4.0)	66.0
Did not complete secondary school	15 (0.6)	53.3	60 (4.5)	83.3
GCSE or GNVQ or equivalent	272 (10.7)	63.2	269 (20.2)	78.4
A-Levels or advanced GNVQ or equivalent	414 (16.2)	66.2	351 (26.3)	77.5
University degree (reference)	874 (34.3)	70.8	386 (28.9)	84.5
Postgraduate degree or higher	759 (29.8)	71.1	215 (16.1)	85.6
PhD	112 (4.4)	68.8	N/A - Not an option	
UK Region				
East Midlands	153 (6.0)	60.8	69 (5.2)	72.5
East of England	269 (10.6)	67.7	174 (13.0)	83.9
London (reference)	464 (18.2)	71.3	266 (19.9)	83.1
North East	76 (3.0)	69.7	32 (2.4)	78.1
North West	275 (10.8)	64.0	93 (7.0)	86.0
Scotland and Northern Ireland	122 (4.8)	62.3	45 (3.4)	73.3
South East	490 (19.2)	72.4	310 (23.2)	80.0
South West	255 (10.0)	71.0	142 (10.6)	81.0
Wales	117 (4.6)	65.0	47 (3.5)	70.2
West Midlands	155 (6.1)	71.6	79 (5.9)	82.3
Yorkshire and The Humber	172 (6.8)	66.3	77 (5.8)	80.5
Prepandemic employment status				
Employed (reference)	1360 (53.4)	66.0	689 (51.6)	82.3
Self-employed	294 (11.5)	68.7	119 (8.9)	80.7
Unemployed	11 (0.4)	63.6	9 (0.7)	100.0
Permanently or long-term sick or disabled	32 (1.3)	53.1	18 (1.3)	44.4
Retired	568 (22.3)	76.4	279 (20.9)	80.3
Other	216 (8.5)	68.5	124 (9.3)	77.4
Unknown	67 (2.6)	62.7	96 (7.2)	81.2
Local area deprivation				
IMD decile 1 (most deprived 10%)	59 (2.3)	47.5	45 (3.4)	75.6
IMD decile 2	97 (3.8)	74.2	68 (5.1)	77.9
IMD decile 3	143 (5.6)	60.1	65 (4.9)	81.5
IMD decile 4	187 (7.3)	66.3	102 (7.6)	81.4
IMD decile 5	209 (8.2)	64.6	133 (10.0)	77.4
IMD decile 6	314 (12.3)	66.9	143 (10.7)	83.2
IMD decile 7	298 (11.7)	68.5	155 (11.6)	81.9
IMD decile 8	356 (14.0)	74.4	188 (14.1)	79.8
IMD decile 9	399 (15.7)	67.9	208 (15.6)	81.2
IMD decile 10 (least deprived 10%) (reference)	486 (19.1)	72.6	227 (17.0)	82.4

Unweighted group sizes and COVID-19 recovery rates among those with self-reported COVID-19 in CSSB and TwinsUK cohorts.

CSSB, COVID Symptom Study Biobank; GCSE, General Certificate of Secondary Education; GNVQ, General National Vocational Qualification; IMD, Index of Multiple Deprivation; PhD, Doctor of Philosophy.

Self-perceived COVID-19 recovery rates were 69% and 81% in CSSB and TwinsUK, respectively, likely reflecting targeted recruitment of individuals with long COVID in CSSB. The Work and Social Adjustment Scale collected for CSSB participants showed high levels of impairment of daily functioning among those who had not recovered from COVID-19 and who self-identified as having or had been diagnosed with long COVID (online supplemental figure 4). A larger proportion of COVID-19 cases were confirmed by self-reported positive antibody or antigen tests (vs suspected or based on medical advice) in CSSB versus TwinsUK (CSSB: 87%, TwinsUK: 71%). At the time of reporting COVID-19 recovery status, most individuals were over a year, and a large proportion over 2 years, since the start of their COVID-19 infection (in CSSB: median: 687 days (IQR: 260–898); in TwinsUK: median: 411 days (IQR: 146–755)). Just over half of COVID-19 cases dated from before the UK vaccination programme commencing in December 2020 (CSSB: 52%, TwinsUK: 51%).

A small number of participants in the CSSB analysis sample were also members of the TwinsUK cohort (34 of 2548, 1.3%), but it was not possible to determine whether these individuals were part of the TwinsUK analysis sample.

### Associations between prepandemic sociodemographics and recovery following COVID-19

Associations between individual sociodemographic variables and recovery following COVID-19 were estimated in Poisson regression models (figure 2, online supplemental tables 1,2). Models included inverse probability weights to account for selection bias from questionnaire response and collider bias from conditioning the analysis sample on COVID-19 infection. Due to the large number of exposure variables tested, we primarily highlight associations that were (1) consistent between cohorts, with (2) effect sizes likely to be meaningful and (3) small uncertainty levels. Whenever results which do not meet these three criteria are described, we state explicitly which criteria were not met.

Estimates consistent across both cohorts, in terms of effect size and CI, were a lower likelihood of recovery for individuals living in the East Midlands region vs London (CSSB: risk ratio (RR) 0.82, 95% CI 0.69 to 0.97, TwinsUK: RR 0.80, 95% CI 0.64 to 1.00) and for those with A-Levels or advanced GNVQ or equivalent level of education (corresponding to a less than degree level, typically finishing education at 18 years old) vs university degree level (CSSB: RR 0.89, 95% CI 0.81 to 0.98, TwinsUK: RR 0.92, 95% CI 0.84 to 1.00). Consistent direction of effect across cohorts but higher uncertainty levels (95% CIs crossing the null) in one of the two cohorts was also seen for age group (positive effect on recovery for ≥70 years old), sex (positive effect on recovery for male sex), employment status (negative effect on recovery for permanently or long-term sick or disabled) and local area deprivation (negative effect for higher deprivation). Effect estimates consistent across cohorts in terms of effect size but with higher uncertainty levels were observed for sex, ethnic group and age group, which showed a weak ‘U-shaped’ trend.

In sensitivity analyses, only marginal changes in effect estimates were seen after inclusion of prepandemic health factors were added as control variables to test an alternative data generation timeline where health factors predominantly precede sociodemographic factors in the causal pathway (online supplemental figure 5). Prepandemic health factors were themselves tested as exposures in secondary analyses (online supplemental figure 6 and tables 1,2).

Additional variables unique to CSSB and TwinsUK cohorts showed further associations (online supplemental figure 7 and tables 1,2). In TwinsUK, lower likelihood of recovery was observed for participants who reported credit or benefit claims prior to the start of the pandemic, or living in homes with damp, mould or vermin at the start of the pandemic.

### Recovery and intersectional social strata

**Figure 3:**
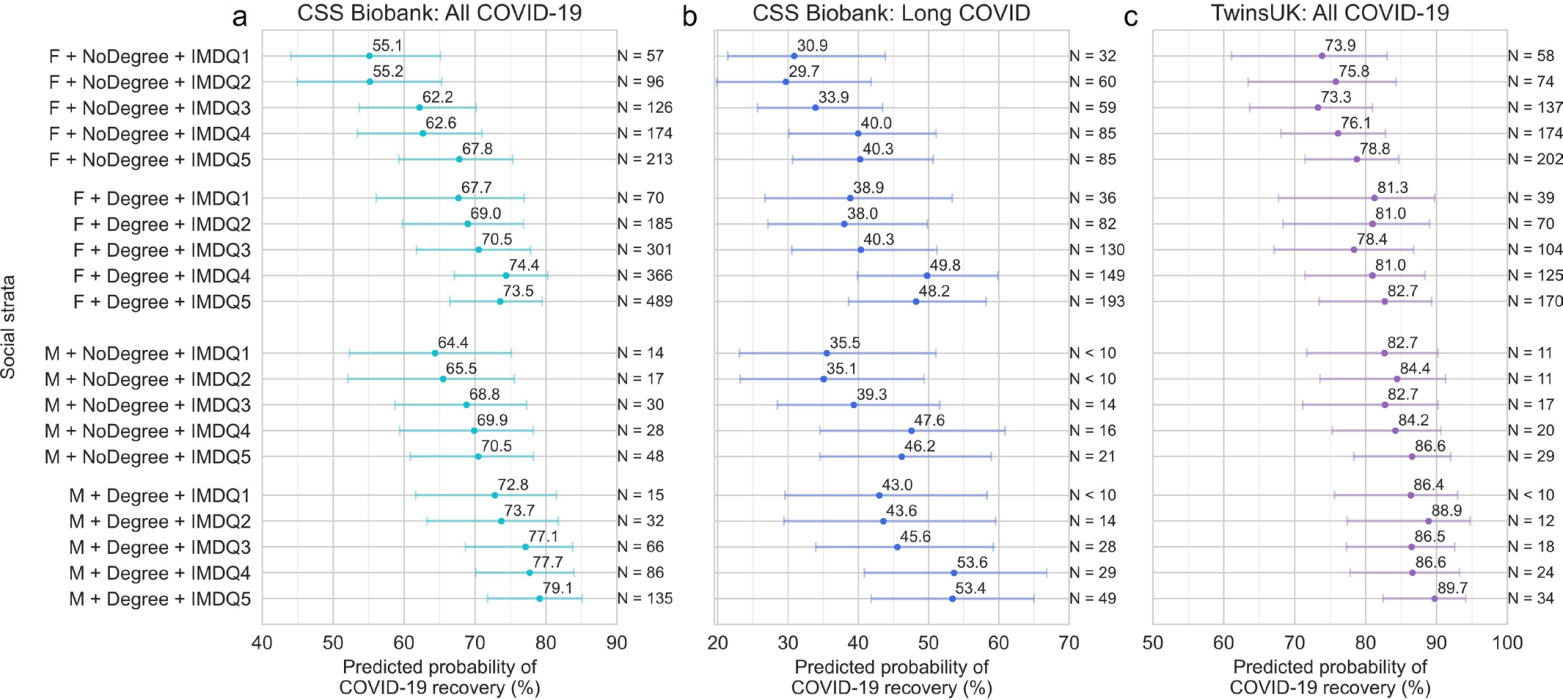
Predicted probability of COVID-19 recovery for social strata of sex, education level and local area deprivation from MAIHDA models in CSS Biobank and TwinsUK cohorts. Predicted probabilities and approximate 95% CIs from MAIHDA mixed-effects logistic regression models testing association between recovery from COVID-19 and social strata for: (a) all CSSB cohort participants with self-reported COVID-19, (b) CSSB participants with diagnosed or self-reported long COVID, (c) all TwinsUK participants with COVID-19. Fixed effects included in models were age group, ethnic group, sex, education level and local area deprivation. Strata labels: F=female, M=male; NoDegree=less than degree level education (including not stated/prefer not to say), Degree=undergraduate degree level or higher; IMDQ1-IMDQ5=Index of Multiple Deprivation Quintile 1–5, where 1 is most deprived 20% of areas, and 5 is least deprived 20%. Models included participant weighting for inverse probability of questionnaire response and selection into analysis samples. CSS Biobank, COVID Symptom Study Biobank; MAIHDA, multilevel analysis of individual heterogeneity and discriminatory accuracy.

In intersectional MAIHDA models estimating associations between COVID-19 recovery and combinations of sex, education level and local area deprivation, female strata consistently had lower predicted probability of COVID-19 recovery than male strata with equivalent education and deprivation level in both cohorts (figure 3, online supplemental tables 3,4). Socioeconomic gradients were observed within female and male strata, with higher probability of recovery observed for strata with higher education and lower deprivation. Among all CSSB participants with COVID-19, there was a 24 percentage point difference between the high education, lowest deprivation male stratum and low education, highest deprivation female stratum (predicted probability=55.1% vs 79.1%) and 16 percentage points in TwinsUK (73.9% vs 89.7%). Patterns were replicated among the subset of CSSB participants with diagnosed or self-reported long COVID (n=1105), with a 22 percentage point difference between the high education, lowest deprivation male stratum and low education, highest deprivation female stratum (53.4% vs 30.9%). Patterns differed slightly between CSSB and TwinsUK, with larger variation with local area deprivation in CSSB than in TwinsUK.

In sensitivity analyses, negligible changes in associations were seen when prepandemic health factors were added as control variables (online supplemental figure 8).

### Associations with sociodemographics during the pandemic

**Figure 4:**
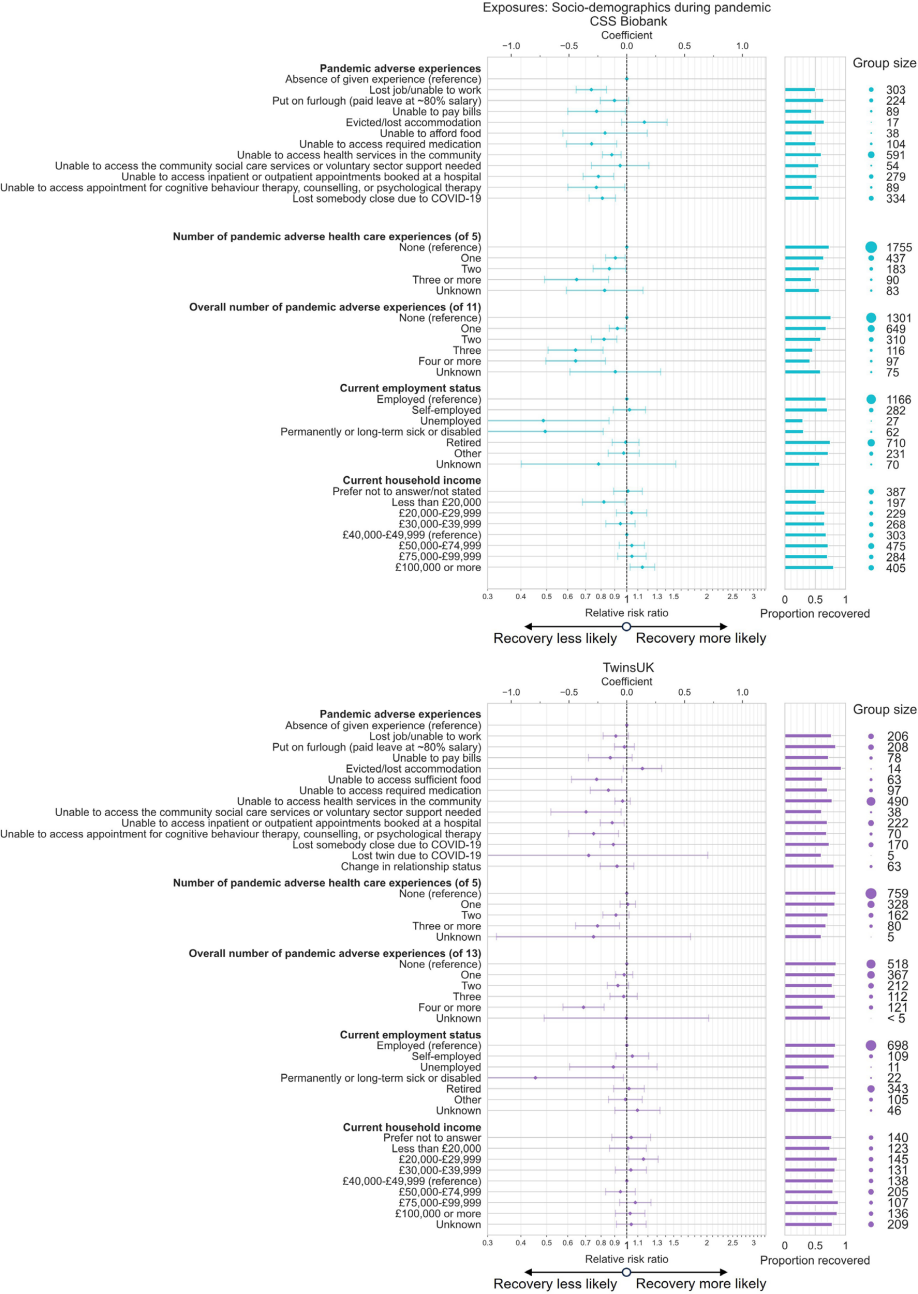
Associations between sociodemographics collected during the COVID-19 pandemic and recovery from COVID-19 in CSS Biobank and TwinsUK cohorts. Relative risk ratio and 95% CIs from Poisson regression models testing association between various sociodemographics collected during the COVID-19 pandemic and recovery from COVID-19, among individuals with self-reported COVID-19 infection. Results for each exposure variable originate from models with distinct adjustment variable sets, including prepandemic sociodemographic factors, prepandemic health characteristics, COVID-19 acute illness factors and sociodemographic factors collected during the pandemic as potential confounding factors as appropriate according to the proposed directed acyclic graph. Models included participant weighting for inverse probability of questionnaire response and selection into the analysis sample. CSS Biobank, COVID Symptom Study Biobank.

Further associations were observed for sociodemographic factors reflecting status or experiences during the pandemic related to housing, employment, finances, access to health and social care and personal relationships (figure 4, online supplemental figure 7 and tables 1,2). For such factors, the directionality of causation was somewhat ambiguous due to unknown temporality of reported experiences relative to COVID-19 illness.

Except for being evicted or losing accommodation, all adverse pandemic experiences (covering employment loss, financial difficulties, housing issues, access to health and social care and bereavement) had negative point estimates in both cohorts, with effect size and uncertainty varying by experience and across cohorts. Composite variables counting the number of adverse health and social care and overall experiences showed dose–response relationships, with strongest negative associations for those with multiple experiences, either for 3 or more of the 5 health and social care related experiences (CSSB: RR 0.65, 95% CI 0.49 to 0.85, TwinsUK: RR 0.78, 95% CI 0.64 to 0.94), or 4 or more from all (11 in CSSB, 13 in TwinsUK) adverse experiences asked about (CSSB: RR 0.64, 95% CI 0.50 to 0.83, TwinsUK: RR 0.69, 95% CI 0.58 to 0.82). Current employment status of permanently or long-term sick or disabled versus employed was also associated with lower likelihood of recovery in both cohorts (CSSB: RR 0.49, 95% CI 0.30 to 0.81, TwinsUK: RR 0.45, 95% CI 0.21 to 0.97).

Models were adjusted for COVID-19 illness characteristics as appropriate according to the proposed DAG and were themselves tested as exposures in secondary analyses (online supplemental figure 9).

## Discussion

### Key points

We found associations between recovery from COVID-19 and explicit combinations of sex, education level and local area deprivation in two different UK longitudinal population studies, CSSB and TwinsUK. Holding multiple advantageous statuses/positions (based on existing systems of power), such as having a degree-level education and living in a less deprived area, was associated with a higher likelihood of reporting full recovery from COVID-19 in both cohorts, in models that accounted for age and ethnic group (figure 3). In contrast, multiple disadvantageous statuses prior to the pandemic were associated with a lower likelihood of recovery and higher rates of ongoing symptoms, on average more than a year since infection. Furthermore, female strata had consistently lower probability of recovery versus male strata of equivalent education and deprivation. Social advantage gradients in recovery rate were not explained by prepandemic health factors (online supplemental figure 8), suggestive of a negligible degree of confounding and/or mediation by prepandemic health factors in the relationship between prepandemic sociodemographic factors and COVID-19 recovery. Results show the importance of considering social determinants in combination rather than in isolation. Non-recovery was also associated with living in housing with mould, damp or vermin issues at the start of the pandemic, as well as adverse employment, financial, health and social care access, and bereavement experiences during the pandemic (figure 4), for which dose–response relationships were observed.

### Interpretation

The observed inequality in recovery from COVID-19 could be theorised as a consequence of structural differences in opportunity and value assigned to certain groups in society,^27^ and/or in terms of differences between groups in social, cultural and economic capital.^46^ Differences in such resources and experiences may affect recovery through the many pathways identified in biopsychosocial models linking socioeconomic status and health more generally.^47^ Previous reports have found associations between long COVID and (in)adequate income, sickness-related absence from work and economic (in)activity,^11 48^ illustrating how ongoing symptoms following COVID-19 may feed into pre-existing negative cycles between low economic capital/poverty and ill health.^49^

We note strong associations between non-recovery and experiences of being unable to access different types of health and social care, which in principle care should be freely available to all within the UK National Health Service but is known to vary by place and person. This is consistent with reports finding that healthcare for COVID-19 has been more accessible for more structurally advantaged groups. Higher likelihood of healthcare disruptions,^50^ and gendered and racialised healthcare experiences,^51^ have been reported for women and racially minoritised ethnic groups in the UK. Furthermore, in conflict with prevalence estimates based on self-report, higher rates of long COVID coding in electronic healthcare records have been found for white ethnic groups and those living in less deprived areas.^52 53^ Other pathways/mechanisms that could be explored in further work include: access to social support networks; inequalities in resources, conditions or responsibilities that affect recuperation, as previously identified^54^,^56^; and biological stressors such as experiences of discrimination including sexism and racism.^57^,^59^ We note that unmeasured confounders such as genetic differences may go further towards explaining the observed social gradient. Equally, there may be more complex interactions between COVID-19 illness and prepandemic health than our models accounted for.

To put our results into context of other illnesses, sociodemographic factors including income, isolation, social support, race/ethnicity, education and local area deprivation have also been found to be social determinants of recovery from mental illness,^60 61^ poor general physical health^62^ and following hospitalisation in critical care.^63^ Such results suggest that social factors likely play a significant role in recovery from illness in general, not only in the case of COVID-19.

### Limitations

We note the limitations in our study. CSSB cohort recruitment was conditioned on the use of a smartphone app, in addition to self-reported SARS-CoV-2 infection status and COVID-19 symptom duration. However, comparable associations were found in TwinsUK, where recruitment was not conditioned on COVID-19 illness. Both cohorts also rely on voluntary participation and analysis conditioned on COVID-19 infection. As such, there is potential for collider bias to operate,^43^ which we attempted to address by using inverse probability weighting in analyses. The biological factor sex was used as a proxy indicator for gender identity as a social determinant of health, due to the absence of gender identity data in both cohorts. In addition to these potential biases, both TwinsUK and CSSB cohorts are overrepresented by white ethnic groups, female sex and those living in more affluent areas when compared with the general UK population, and so results may not be generalisable to the whole UK population. CSSB participants with asymptomatic infection (N=140) were mistakenly not asked the COVID-19 recovery question, given that long COVID is known to occur among those with asymptomatic infection^64^ and thus not included in analyses.

Inclusion of certain factors in intersectional MAIHDA analysis with groups known to be subject to structural social disadvantage was limited by sample size, such as small numbers of distinct racially minoritised ethnic groups, or not possible due to absence of data, such as for LGBTQ+ groups. Variables included in social strata were thus limited to those with large group sizes and required aggregation of some categories. While we included potential confounding variables as fixed effects in MAIHDA models, following a causal inference approach, we note that the variables in the social strata cover large time ranges (ie, sex at birth to deprivation of local area prior to the COVID-19 pandemic), which increases the likelihood of violating the no intermediate confounding assumption of the causal inference framework.

### Summary

In summary, our analyses suggest there is a health gradient in self-perceived recovery from ongoing symptoms following COVID-19 infection along the lines of structural societal advantage. Recovery correlates strongly with functional impairment. COVID-19 may be a model illness for demonstrating social inequalities in recovery more generally. Further research could assess whether disparities in recovery have contributed to a widening of health inequalities during the COVID-19 pandemic for socially disadvantaged groups, or rather represent a continuation of pre-existing inequalities. Targeted support for individuals during recovery periods from acute illness may help to address inequalities, informed by further investigation of the biopsychosocial mechanisms that underlie social inequalities in recovery following COVID-19 as well as other illnesses.

### Plain language summary

Across the world, acute COVID-19 illness has affected the most disadvantaged in society the most. However, we have not looked in detail whether people’s social circumstances affect their recovery from COVID-19. In our study, we asked people from two UK-based health studies if they still had symptoms after having COVID-19. We looked at how advantaged or disadvantaged they were at the start of the pandemic, based on information about their sex, education level and their local area. In both studies, people who were more disadvantaged were more likely to still have symptoms long after having COVID-19. In contrast, more advantaged people were more likely to feel fully recovered. We also saw that people who had multiple negative experiences during the pandemic, such as losing their job, being unable to afford their bills, losing a loved one or not being able to access health and social care services were less likely to recover. More work is needed to understand how and why recovery was so different for people with different circumstances. Our study suggests that health policies need to address multiple aspects of people’s lives at the same time to improve health.

## Supplementary material

10.1136/bmjph-2024-001166 Supplementary file 1

## Data Availability

Data are available on reasonable request.
