# Supplementary material for: Social determinants of recovery from ongoing symptoms following COVID-19 in two UK longitudinal studies: a prospective cohort study
Source: BMJ Public Health. 2025 Mar 20;3(1):e001166. doi: 10.1136/bmjph-2024-001166 (PMC12007038; doi:10.1136/bmjph-2024-001166)
Supplement: Supplementary file 1 [file bmjph-3-1-s001.pdf]

Social determinants of recovery from ongoing symptoms following COVID-19 in two UK longitudinal studies: a prospective cohort study

Supplementary information

Authors & affiliations

Nathan J. Cheetham[1]*, Vicky Bowyer[1], María Paz García[1], Ruth C. E. Bowyer[1,2], J. D. Carpentieri[3], Andy Guise[4], Ellen J. Thompson[1,5], Carole H. Sudre[6,7,8], Erika Molteni[8], Michela Antonelli[8], Rose S. Penfold[9], Nicholas R. Harvey[1], Liane S. Canas[8], Khaled Rjoob[6], Benjamin Murray[8], Eric Kerfoot[8], The COVID Symptom Study Biobank Consortium[^], Alexander Hammers[8,10], Sebastien Ourselin[8], Emma L. Duncan[1,11], Claire J. Steves[1,11]*

* Corresponding authors

Correspondence to: nathan.cheetham@kcl.ac.uk claire.j.steves@kcl.ac.uk

1 Department of Twin Research and Genetic Epidemiology, King's College London, London, United Kingdom

2 The Alan Turing Institute, London, United Kingdom

3 Institute of Education, University College London, London, United Kingdom

4 Department of Population Health Sciences, King's College London, London, United Kingdom

5 School of Psychology, University of Sussex, Falmer, United Kingdom

6 MRC Unit for Lifelong Health and Ageing, Department of Population Science and Experimental Medicine, University College London, London, United Kingdom

7 Centre for Medical Image Computing, Department of Computer Science, University College London, London, United Kingdom

8 School of Biomedical Engineering & Imaging Sciences, King's College London, London, UK

9 Edinburgh Delirium Research Group, Ageing and Health, Usher Institute, University of Edinburgh, Edinburgh, United Kingdom

10 King's College London & Guy's and St Thomas' PET Centre, King's College London, London, United Kingdom

11 Guy’s & St Thomas’s NHS Foundation Trust, London, United Kingdom

^A list of authors and their affiliations appears below

The COVID Symptom Study Biobank Consortium

Michela Antonelli[1], Vicky Bowyer[2], Julia Brown[2,3], Liane Canas[1], Joan Capdevila Pujol[4], Nathan Cheetham[2], Lynn Cherkas[2], Jie Deng[1], Katie Doores[5], Emma Duncan[2,6], Maria Paz Garcia[2], Alexander Hammers[1,7], Deborah Hart[2], Nicholas Harvey[2], Adrian Hopper[8], Christina Hu[4], Eric Kerfoot[1], Michael Malim[5], Marc Modat[1], Erika Molteni[1], Benjamin Murray[1], Ayrun Nessa[2], Sebastien Ourselin[1], Tim Spector[2], Claire Steves[2,9], Carole Sudre[1,10,11], Samuel Wadge[2], Jonathan Wolf[4]

1 School of Biomedical Engineering & Imaging Sciences, King’s College London, London, United Kingdom.

2 Department of Twin Research and Genetic Epidemiology, King’s College London, London, United Kingdom.

3 Medical Student, James Cook University, Australia.

4 Zoe Ltd, 164 Westminster Bridge Road, London, United Kingdom.

5 Department of Infectious Diseases, King’s College London, London, United Kingdom.

6 Department of Endocrinology, Guy’s and St Thomas’ NHS Foundation trust, London, United Kingdom.

7 King's College London & Guy's and St Thomas' PET Centre, King's College London, London, United Kingdom.

8 Guy’s and St Thomas’ NHS Foundation trust, London, United Kingdom.

9 Department of Ageing and Health, Guy’s and St Thomas’ NHS Foundation trust, London, United Kingdom.

10 MRC Unit for Lifelong Health and Ageing, Department of Population Science and Experimental Medicine, University College London, London, United Kingdom.

11 Centre for Medical Image Computing, Department of Computer Science, University College London, London, United Kingdom.

Data collection timeline

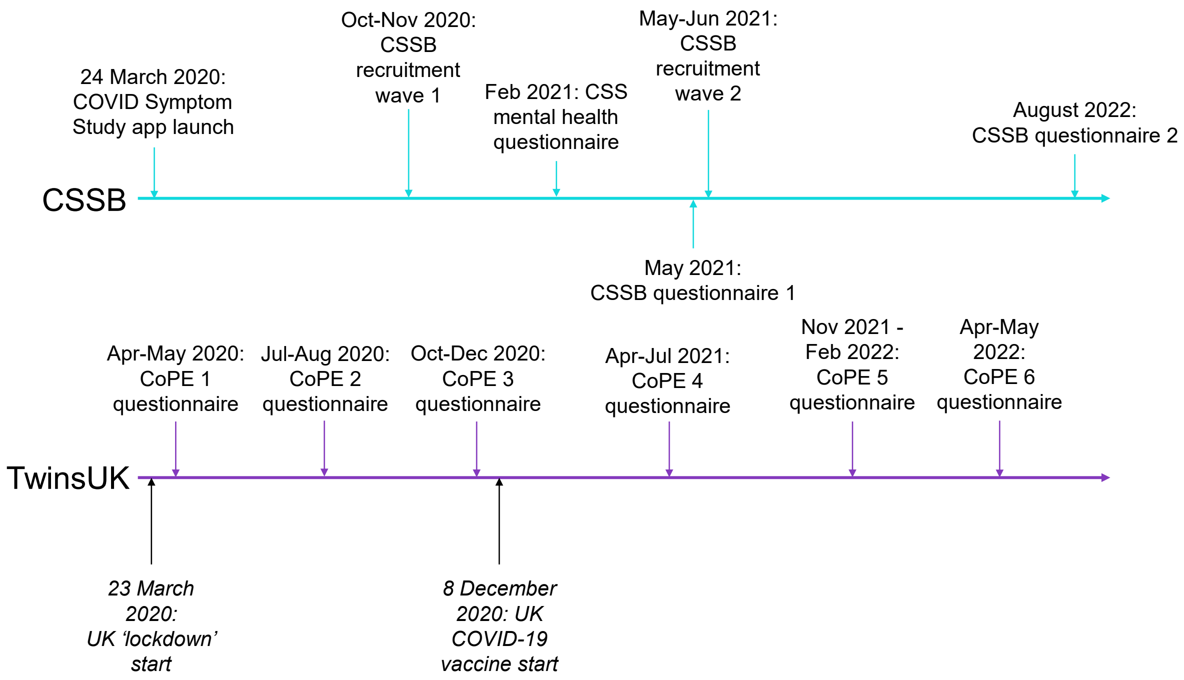

Figure S 1. **Timeline of data collections for CSSB and TwinsUK.** CSS: COVID Symptom Study app, CSSB: COVID Symptom Study Biobank.

1. CSSB Data collection

Socio-demographic characteristics

Information on age group at the time of the 2022 CSSB questionnaire was derived from date of birth self-reported at CSSB consent (2022 – Year of Birth).

Information on sex assigned at birth (female/intersex/male/prefer not to say) was self-reported at registration to the CSS app.

Information on race/ethnicity was collected at CSSB consent with the question "what is your ethnic group?", using UK 2021 census categories (Asian/Asian British, Black/Black British/Caribbean/African, Mixed/Multiple ethnic groups, and Other ethnic groups) [1].

First language was self-reported in the August, 2022 CSSB questionnaire, and grouped into English and Other due to small number of individuals with first languages other than English.

Information on highest educational qualification was collected in the August, 2022 CSSB questionnaire "What is the highest academic/educational qualification (or its nearest equivalent) you have received?".

Local area deprivation, UK geographic region of residence and Rural-Urban classification (most recent version from each UK nation) were derived from address data collected upon joining the CSS app and CSSB consent (precedence given to earlier CSS app data). Deprivation was measured by the Index of Multiple Deprivation (IMD) quantiles of lower super output area rank, with data from England 2019 [2], Wales 2019 [3], Scotland 2020 [4], and Northern Ireland 2017 [5]. Individuals living in Scotland and Northern Ireland were grouped together in “Scotland/Northern Ireland” category, due to small number of participants residing in Northern Ireland. Scotland was chosen as the geographically closest other UK region.

Equivalent data for education level and residential address immediately prior to the COVID-19 pandemic was not available and responses were assumed to represent pre-pandemic statuses.

Information on pre-pandemic employment status was self-reported in the May, 2021 CSSB questionnaire in the question “Which one of these best describes what you were doing before the COVID-19 pandemic? If you were doing more than one activity, please choose the activity you spent the most time doing”. Due to small numbers of individuals, “Other” was a heterogeneous group comprising “In unpaid/voluntary work”, “In education at school/college/university, or in an apprenticeship”, “Looking after home or family”, “Other” options.

Current employment status was self-reported in the August, 2022 CSSB questionnaire, with “other” options grouped as for the pre-pandemic equivalent.

Current yearly gross household income was self-reported in the August, 2022 CSSB questionnaire with the question “What is the total yearly income before tax received by your household? This includes all those who can earn and live in the same household as yourself.”

Adverse experiences during the pandemic were measured in the August, 2022 CSSB questionnaire with the question: “Have you experienced any of the following during the COVID-19 pandemic?” (Yes/No/Prefer not to say). There were 11 experiences that respondents could report covering employment, housing, finances, health & social care access and bereavement. The number of general health and social care access issues experienced during the pandemic was generated from the number of Yes responses to the 5 response options: “Unable to access required medication”, “Unable to access health services in the community, for instance, GP/community physiotherapy/nurse/podiatrist/dentist”, “Unable to access the community social care services or voluntary sector support you need, for instance, from carers or day centres”, “Unable to access inpatient or outpatient appointments booked at a hospital for a consultation, investigation, treatment, or surgery”, and “Unable to access appointment for cognitive behaviour therapy, counselling, or psychological therapy”.

Health characteristics

Pre-pandemic general health was self-reported in the May, 2021 CSSB questionnaire with the question “In general, in the 3 months before the COVID-19 outbreak in March 2020, would you say your health was...”.

Body mass index (BMI) was derived from self-reported height and weight collected at CSSB consent.

Frailty, a measure of age-related decline in physiological reserve and function [6], was measured using the PRISMA-7 scale [7], collected at registration with the CSS app.

Number of physical health conditions was measured from 6 self-reported conditions (asthma, cancer, diabetes, heart disease, lung disease, kidney disease) collected at registration for the CSS app.

Number of mental health conditions was measured from self-reported diagnoses of 16 conditions (Generalised anxiety order; Panic disorder; Specific phobias; Obsessive compulsive disorder; Post-traumatic stress disorder; Social anxiety disorder; Agoraphobia; Depression; Attention deficit or attention deficit and hyperactivity disorder; Autism, Asperger's or autistic spectrum disorder; Eating disorder (e.g. bulimia nervosa; anorexia nervosa; psychological over-eating or binge-eating), Personality disorder; Mania, hypomania, bipolar or manic depression; Schizophrenia; Substance use disorder; Any other type of psychosis or psychotic illness) collected in a February, 2021 CSS questionnaire.

Equivalent data used to derive BMI, frailty and number of physical health conditions representing status immediately prior to the COVID-19 pandemic was not available and responses were assumed to represent pre-pandemic statuses.

Work and social adjustment scale (WSAS) [8], collected in the August, 2022 CSSB questionnaire, asked individuals to assess how their health impaired their ability to do day-to-day tasks over the last 3 months.

COVID-19 illness characteristics

COVID-19 infection history was measured with the question “How many times do you think you have ever had COVID-19? Please include now if you think you currently have COVID-19 symptoms but have not confirmed it.”. For each reported infection, participants were asked what evidence supported their infection (“How do/did you know you had it (COVID-19)?”), the start date of the infection/illness (“When do you think you had COVID-19? Please use your best estimate if you can't remember the exact date.”), the duration of COVID-19 symptoms (“How long did you have or have you had continuous symptoms?”) and the duration for which they were not able to function as normal (“How long were you, or have you been unable to function as normal due to COVID-19 symptoms?”). Where multiple infections were reported, information was obtained for the single

infection with the longest reported symptom duration, then earliest date of infection start, then strongest evidence of infection.

In addition to retrospective self-reported symptom duration, prospective symptom reporting was available for CSSB participants via self-reporting using the CSS app. Symptom duration from prospective symptom reporting in the app was estimated using methods described in previous reports [9].

Infection period was derived from self-reported date of infection start. Periods were defined based on changes in dominant SARS-CoV-2 variant from COG-UK Mutation Explorer data available at https://sars2.cvr.gla.ac.uk/cog-uk/.

Whether individual accessed urgent care during their COVID-19 illness was assessed from the questions "What type of medical help did you access WITHIN the first 4 weeks of (/MORE THAN 4 weeks after) the start of your symptoms that you think may have been caused by COVID-19?", from selection of options "Visited A&E or walk-in centre" or "Called an ambulance".

Number of new conditions due to COVID-19 was generated from responses to the question "Please tell us which new health condition, illness, or disability you have been told you have since March 2020.", from 22 health condition options, where individuals selected Yes to "Has a doctor told you that this new health condition developed because of COVID-19 infection?".

Self-reported or diagnosed long COVID was measured by the question "Have you ever received a diagnosis of long COVID or post-COVID syndrome?", with "No, but I do believe I have or have had Long COVID" or "Yes" taken as indicative of long COVID and "No, and I do not believe I have or have had Long COVID" as not.

2. TwinsUK Data collection

Socio-demographic characteristics

Information on sex assigned at birth and age group at time of latest questionnaire (age calculated as 2022 – Year of Birth) from date of birth was collected at TwinsUK registration. Information on race/ethnicity and highest educational qualification was collected as part of regular TwinsUK longitudinal questionnaires.

Local area deprivation, UK geographic region of residence and Rural-Urban classification (most recent version from each UK nation) were derived from most recent address data as of April 1, 2020.

Information on pre-pandemic employment status was self-reported in CoPE #1, #4 and #5 questionnaires, and current employment status collected in CoPE #4, #5 and #6, using the same question and response options as described for CSSB. Earliest and latest valid responses were taken for pre-pandemic and current employment status respectively.

Yearly gross household income was collected as part of TwinsUK longitudinal questionnaires. Pre-pandemic income was generated from the latest available response between January 1, 2017 and April 1, 2020, while latest income considered the latest available response after January 1, 2017.

Adverse experiences during the pandemic were measured in every CoPE #1 to #6, with the question: "Have you experienced any of the following as a result of COVID-19?". Response options were expanded over earlier CoPE rounds based on increased understanding of adverse experiences, to be equivalent to CSSB from CoPE #4 onwards. Responses in TwinsUK were treated as equivalent to CSSB, and interpreted as experiences during the COVID-19 pandemic in both cases, despite differences in question wording. For responses across multiple questionnaires, the maximal responses were taken.

Variables unique to TwinsUK:

Housing tenure and housing problems with damp, mould or vermin were self-reported in CoPE #1.

Credit or benefits claims prior to the pandemic was collected in CoPE #1 and #6 with the question "Before the pandemic (March 2020), did you or your partner regularly claim for the following?", with Yes/No for the following response options: Free school meals, Universal credit, Pension credit, Employment Support allowance, Statutory sick pay, Housing benefit, council tax benefit, carers allowance and Personal Independence Payment (PIP). New credit or benefits claims during the pandemic was collected in CoPE #2, #3 and #6 with the question "Since the start of the pandemic (March 2020), have you or your partner ever made any new claims for the following?", with the same 6 response options as the pre-pandemic question, plus the additional option "A grant through the new self-employment income support scheme" which described the UK government scheme to cover

wages for those whose work was affected by the COVID pandemic. For responses across multiple questionnaires, the maximal responses were taken.

Number of significant stressors was derived from the following question asked in CoPE #1 and #2: "Have any of these things been causing you SIGNIFICANT stress? E.g. They have been constantly on your mind or have been keeping you awake at night", counting the number of 'Yes' responses to the following 16 response options: Marriage or other romantic relationship, Friends or family living in your household, Friends or family living outside your household, Neighbours, Your pet(s), Work (even if you feel your job is safe), Losing your job/unemployment, Finances, Getting medication, Getting food, Your own safety/security, Internet access, Boredom, Future plans, Catching COVID-19, Becoming seriously ill from COVID-19. For responses across multiple questionnaires, the maximal responses were taken.

Number of caring responsibilities was derived from the following questions asked in CoPE #1, #2 and #3: "Are you currently responsible for the care or support of any of the following INSIDE the home?" and "Are you currently responsible for the care or support of any of the following OUTSIDE the home?", counting the number of 'Yes' responses to the following response options which were asked in both questions: Elderly relatives or friends, People with long-term conditions or disabilities, Grandchildren, Children (under 18). For responses across multiple questionnaires, the maximal responses were taken.

Health characteristics

Pre-pandemic general health was self-reported in CoPE #1 and #2 questionnaires and was the same as described in CSSB. For responses across multiple questionnaires, the earliest valid response was taken.

Body mass index (BMI) was derived from latest available height and weight data prior to April 1, 2020, collected from either self-report in regular longitudinal questionnaires or clinic visits.

Frailty, measured using the PRISMA-7 scale, was collected in CoPE #1 and #2 questionnaires. For responses across multiple questionnaires, the earliest valid response was taken.

Number of physical health and mental health conditions were collected from latest available responses in regular longitudinal questionnaires. The same physical conditions as collected in CSSB were chosen for cross-cohort harmonisation. Mental health conditions available in TwinsUK were limited to 4 conditions: bipolar disorder, anxiety/stress disorder, depression and eating disorder.

COVID-19 illness characteristics

COVID-19 infection history was derived based on self-report in each of CoPE #1 to #6. As in CSSB, information was collected on evidence of infection, start date of infection/illness, duration of COVID-19 symptoms, and duration for which they were not able to function as normal. Where multiple

infections were reported, information was obtained for the single infection with the longest reported symptom duration, then earliest date of infection start, then strongest evidence of infection.

Infection period was derived from self-reported date of infection start as in CSSB. COVID-19 illness acute severity was measured in TwinsUK through questions on whether individuals were admitted to hospital as a result of COVID-19, asked in CoPE #2 through #6. For responses across multiple questionnaires, the maximal responses were taken. Number of new conditions due to COVID-19 asked to CSSB participants was not available in TwinsUK.

Self-reported or diagnosed long COVID was measured using the same question as described in CSSB, asked in CoPE #5 and #6. For responses across multiple questionnaires, the latest valid response was taken. We note however, that the first iteration of the question in CoPE #5 had only "Yes" and "No" as response options, leaving individuals' self-perception of whether they had long COVID unspecified. Therefore, an additional category, "No (self-reported long COVID status unknown)" was included in TwinsUK analyses for responses based on this question.

Sample selection flow diagram

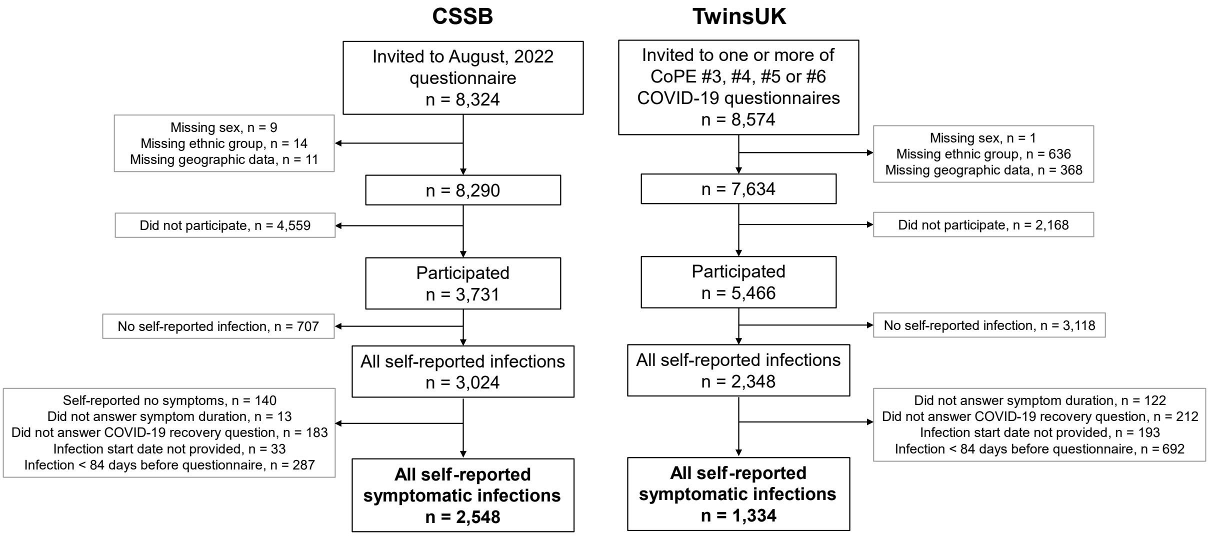

Figure S 2. **Sample selection flow diagram.** Exclusions are identified in grey boxes.

Full directed acyclic graph

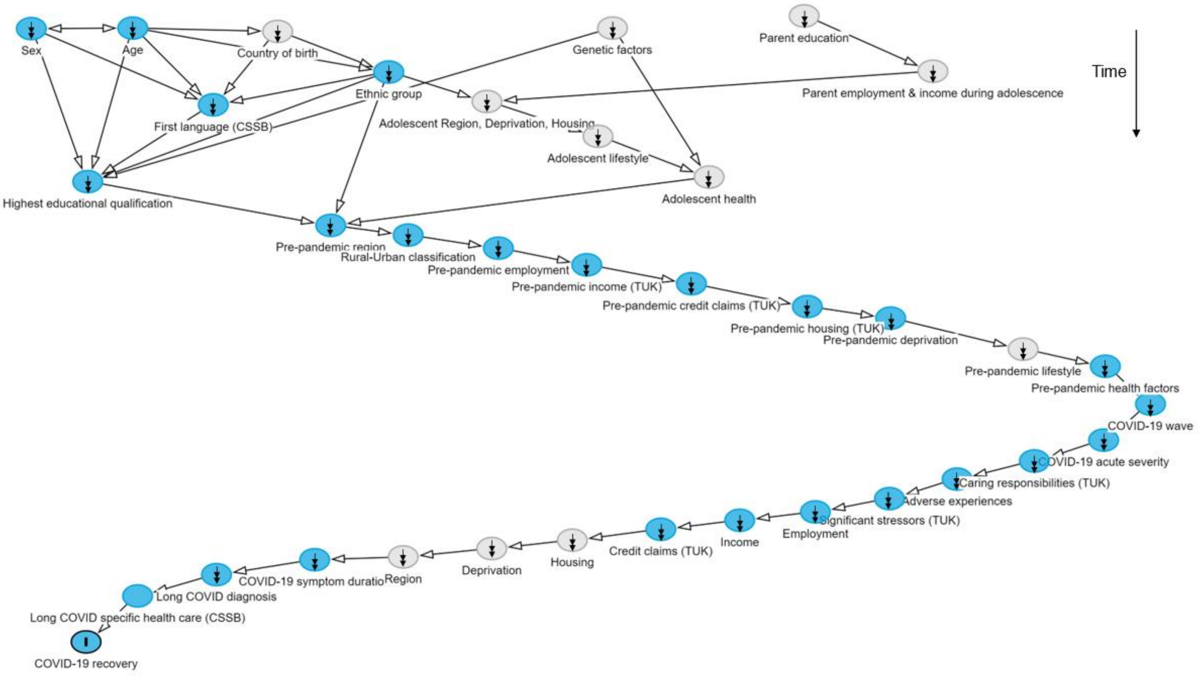

Figure S 3. **Full directed acyclic graph describing hypothesised causal pathways**. Proposed directed acyclic graph (DAG) used to generate minimal adjustment variable sets for models estimating the total causal effect of exposure variables on the outcome of self-perceived COVID-19 recovery. For clarity, only key 'nearest neighbour' arcs are drawn, and double headed arrows within variables are used to represent where variables are theorised to affect all following variables (i.e. arcs present to all following variables). Data only available in CSSB or TwinsUK is noted in brackets, while key unobserved potential confounders are coloured in grey. The DAG is structured approximately in order of data generation/crystallisation from top to bottom.

Extended sample characteristics & multivariable poisson regression results

COVID Symptom Study Biobank

Table S 1. **Extended sample characteristics and results of multivariable poisson regression models, CSSB cohort.** Relative risk ratios, 95% confidence intervals (CI) and p-values adjusted for multiple testing (Benjamini/Hochberg false discovery rate correction) are presented for multivariable poisson regression models testing association between recovery from COVID-19 and exposure of interest, after adjustment as appropriate from the hypothesised directed acyclic graph (DAG), and weighting for inverse probability of questionnaire participation and selection into analysis sample.

		COVID Symptom Study Biobank (CSSB)					
Domain	Variable	Group size, N (%)	COVID-19 recovery (%)	Relative risk ratio	95% CI (lower)	95% CI (upper)	P-value (adjusted)
**Individual pre-pandemic demographics**	**Age group (years)**						
	18-39	155 (6.1%)	71.6%	1.09	0.96	1.24	0.290
	40-49	386 (15.1%)	63.5%	1.02	0.92	1.12	0.759
	50-59 (reference)	850 (33.4%)	65.4%	1.00			
	60-69	873 (34.3%)	71.1%	1.06	0.99	1.15	0.203
	≥ 70	284 (11.1%)	75.7%	1.12	1.01	1.24	0.058
	**Sex**						
	Female (reference)	2077 (81.5%)	67.6%	1.00			
	Male	471 (18.5%)	72.8%	1.07	0.99	1.15	0.132
	**Ethnic group**						
	Asian/Asian British	11 (0.4%)	54.5%	0.80	0.44	1.46	0.574
	Black/Black British	7 (0.3%)	71.4%	1.12	0.77	1.63	0.659
	Mixed/Multiple	22 (0.9%)	68.2%	0.79	0.51	1.23	0.415
	Other	32 (1.3%)	50.0%	0.81	0.57	1.15	0.350
	White (reference)	2476 (97.2%)	68.9%	1.00			
	**Highest educational qualification**						
	Prefer not to answer/not stated	102 (4.0%)	56.9%	0.70	0.48	1.02	0.126
	Did not complete secondary school	15 (0.6%)	53.3%	0.68	0.39	1.18	0.272
	GCSE or GNVQ or equivalent	272 (10.7%)	63.2%	0.86	0.76	0.97	0.032
	A-Levels or advanced GNVQ or equivalent	414 (16.2%)	66.2%	0.89	0.81	0.98	0.050
	University degree (reference)	874 (34.3%)	70.8%	1.00			
	Postgraduate degree or higher	759 (29.8%)	71.1%	1.00	0.94	1.07	0.952
	PhD	112 (4.4%)	68.8%	0.94	0.81	1.10	0.545
	**UK Region**						
	East Midlands	153 (6.0%)	60.8%	0.82	0.69	0.97	0.054
	East of England	269 (10.6%)	67.7%	0.93	0.82	1.05	0.351
	London (reference)	464 (18.2%)	71.3%	1.00			
	North East	76 (3.0%)	69.7%	0.96	0.80	1.16	0.738
	North West	275 (10.8%)	64.0%	0.92	0.81	1.03	0.260

	Scotland & Northern Ireland	122 (4.8%)	62.3%	0.87	0.72	1.04	0.206
	South East	490 (19.2%)	72.4%	1.06	0.98	1.16	0.259
	South West	255 (10.0%)	71.0%	0.97	0.87	1.09	0.716
	Wales	117 (4.6%)	65.0%	0.96	0.82	1.11	0.660
	West Midlands	155 (6.1%)	71.6%	0.92	0.79	1.08	0.434
	Yorkshire and The Humber	172 (6.8%)	66.3%	0.93	0.81	1.07	0.452
	**Rural-Urban classification**						
	Rural	600 (23.5%)	72.3%	1.11	1.03	1.18	0.013
	Urban (reference)	1948 (76.5%)	67.5%	1.00			
	**Pre-pandemic employment status**						
	Employed (reference)	1360 (53.4%)	66.0%	1.00			
	Self-employed	294 (11.5%)	68.7%	0.98	0.88	1.08	0.716
	Unemployed	11 (0.4%)	63.6%	0.87	0.53	1.44	0.684
	Permanently or long-term sick or disabled	32 (1.3%)	53.1%	0.69	0.45	1.05	0.155
	Retired	568 (22.3%)	76.4%	1.07	0.98	1.17	0.242
	Other	216 (8.5%)	68.5%	1.02	0.91	1.14	0.783
	Unknown	67 (2.6%)	62.7%	0.96	0.79	1.16	0.716
	**Local area deprivation**						
	IMD Decile 1 (most deprived 10%)	59 (2.3%)	47.5%	0.60	0.42	0.87	0.020
	IMD Decile 2	97 (3.8%)	74.2%	1.10	0.95	1.27	0.318
	IMD Decile 3	143 (5.6%)	60.1%	0.87	0.74	1.01	0.138
	IMD Decile 4	187 (7.3%)	66.3%	0.90	0.79	1.03	0.219
	IMD Decile 5	209 (8.2%)	64.6%	0.89	0.79	1.01	0.152
	IMD Decile 6	314 (12.3%)	66.9%	0.93	0.84	1.04	0.294
	IMD Decile 7	298 (11.7%)	68.5%	0.89	0.79	1.00	0.113
	IMD Decile 8	356 (14.0%)	74.4%	1.01	0.92	1.12	0.808
	IMD Decile 9	399 (15.7%)	67.9%	0.95	0.87	1.04	0.412
	IMD Decile 10 (least deprived 10%) (reference)	486 (19.1%)	72.6%	1.00			
**Socio-demographics during pandemic**	**Pandemic adverse experiences**						
	Absence of given experience (reference)			1.00			
	Lost job/unable to work	303 (11.9%)	50.2%	0.74	0.65	0.84	0.000
	Put on furlough (paid leave at ~80% salary)	224 (8.8%)	63.8%	0.90	0.80	1.02	0.158
	Unable to pay bills	89 (3.5%)	43.8%	0.77	0.60	0.99	0.081
	Evicted/lost accommodation	17 (0.7%)	64.7%	1.16	0.96	1.42	0.221
	Unable to afford food	38 (1.5%)	44.7%	0.83	0.58	1.19	0.431
	Unable to access required medication	104 (4.1%)	51.0%	0.74	0.59	0.92	0.019
	Unable to access health services in the community	591 (23.2%)	60.1%	0.88	0.81	0.95	0.007
	Unable to access the community social care services or voluntary sector support needed	54 (2.1%)	55.6%	0.94	0.74	1.21	0.716

	Unable to access inpatient or outpatient appointments booked at a hospital	279 (10.9%)	52.7%	0.78	0.69	0.89	0.002
	Unable to access appointment for cognitive behaviour therapy, counselling, or psychological therapy	89 (3.5%)	44.9%	0.77	0.60	0.98	0.075
	Lost somebody close due to COVID-19	334 (13.1%)	56.3%	0.81	0.72	0.91	0.002
	**Number of pandemic adverse health care experiences (of 5)**						
	None (reference)	1755 (68.9%)	72.9%	1.00			
	One	437 (17.2%)	63.8%	0.91	0.83	0.99	0.054
	Two	183 (7.2%)	56.8%	0.86	0.75	0.99	0.075
	Three or more	90 (3.5%)	43.3%	0.65	0.49	0.85	0.008
	Unknown	83 (3.3%)	56.6%	0.83	0.59	1.15	0.366
	**Overall number of pandemic adverse experiences (of 11)**						
	None (reference)	1301 (51.1%)	75.9%	1.00			
	One	649 (25.5%)	67.8%	0.92	0.86	0.99	0.054
	Two	310 (12.2%)	59.0%	0.82	0.74	0.92	0.002
	Three	116 (4.6%)	45.7%	0.64	0.51	0.81	0.002
	Four or more	97 (3.8%)	41.2%	0.64	0.50	0.83	0.004
	Unknown	75 (2.9%)	58.7%	0.91	0.61	1.34	0.704
	**Current employment status**						
	Employed (reference)	1166 (45.8%)	67.6%	1.00			
	Self-employed	282 (11.1%)	69.9%	1.02	0.89	1.18	0.789
	Unemployed	27 (1.1%)	29.6%	0.49	0.27	0.86	0.034
	Permanently or long-term sick or disabled	62 (2.4%)	30.6%	0.49	0.30	0.81	0.019
	Retired	710 (27.9%)	74.8%	0.99	0.88	1.11	0.881
	Other	231 (9.1%)	71.4%	0.97	0.85	1.11	0.749
	Unknown	70 (2.7%)	57.1%	0.78	0.40	1.53	0.574
	**Current household income**						
	Prefer not to answer/not stated	387 (15.2%)	65.4%	1.01	0.89	1.14	0.881
	Less than £20,000	197 (7.7%)	51.8%	0.82	0.68	0.99	0.075
	£20,000-£29,999	229 (9.0%)	65.5%	1.04	0.91	1.19	0.638
	£30,000-£39,999	268 (10.5%)	65.3%	0.95	0.83	1.07	0.508
	£40,000-£49,999 (reference)	303 (11.9%)	68.0%	1.00			
	£50,000-£74,999	475 (18.6%)	71.2%	1.04	0.94	1.16	0.545
	£75,000-£99,999	284 (11.1%)	70.1%	1.04	0.92	1.18	0.582
	£100,000 or more	405 (15.9%)	80.2%	1.14	1.03	1.27	0.034
**Pre-pandemic health factors**	**Pre-pandemic health**						
	Poor	65 (2.6%)	55.4%	0.90	0.70	1.15	0.508
	Fair	179 (7.0%)	54.2%	0.85	0.74	0.98	0.058
	Good	553 (21.7%)	63.3%	0.92	0.84	1.00	0.120

	Very good (reference)	922 (36.2%)	70.5%	1.00			
	Excellent	496 (19.5%)	76.6%	1.09	1.01	1.17	0.061
	Unknown	333 (13.1%)	70.6%	1.06	0.97	1.16	0.329
	**Body mass index**						
	< 18.5 kg/m^2	32 (1.3%)	68.8%	0.96	0.74	1.25	0.808
	18.5-25 kg/m^2 (reference)	1112 (43.6%)	74.6%	1.00			
	25-30 kg/m^2	808 (31.7%)	66.7%	0.89	0.83	0.96	0.008
	≥ 30 kg/m^2	591 (23.2%)	59.7%	0.82	0.76	0.90	1E-04
	**PRISMA-7 Frailty score**						
	0-2, below threshold (reference)	2422 (95.1%)	69.7%	1.00			
	3-7, above threshold	126 (4.9%)	48.4%	0.74	0.60	0.90	0.011
	**Number of physical health conditions**						
	None (reference)	1958 (76.8%)	71.0%	1.00			
	One	515 (20.2%)	62.5%	0.92	0.85	0.99	0.069
	Two or more	75 (2.9%)	46.7%	0.70	0.53	0.92	0.029
	**Number of mental health conditions (February 2021)**						
	None (reference)	1534 (60.2%)	74.1%	1.00			
	One	360 (14.1%)	60.0%	0.86	0.78	0.95	0.011
	Two	129 (5.1%)	59.7%	0.93	0.80	1.09	0.494
	Three or more	58 (2.3%)	48.3%	0.88	0.68	1.15	0.460
	Unknown	467 (18.3%)	62.3%	0.89	0.81	0.97	0.018
	4 to 12 weeks	330 (13.0%)	63.0%	0.79	0.72	0.86	7E-06
**COVID-19 illness characteristics**	**Infection period**						
	Before 2020-12-08 (pre-vaccination, wild-type dominant) (reference)	1458 (57.2%)	64.7%	1.00			
	2020-12-08 to 2021-04-25 (alpha-variant dominant)	268 (10.5%)	51.9%	0.84	0.74	0.96	0.034
	2021-04-25 to 2021-12-08 (delta-variant dominant)	142 (5.6%)	73.9%	1.09	0.96	1.25	0.290
	After 2021-12-08 (omicron-variant dominant)	680 (26.7%)	82.4%	1.24	1.17	1.32	5E-11
	**Urgent care accessed during COVID-19 illness**						
	No (reference)	2316 (90.9%)	71.5%	1.00			
	Yes	232 (9.1%)	39.7%	0.60	0.49	0.73	7E-06
	**Symptom duration (retrospective self-report)**						
	Less than 2 weeks (reference)	966 (37.9%)	88.2%	1.00			
	2 to 4 weeks	581 (22.8%)	74.9%	0.88	0.83	0.94	0.001
	3 to 6 months	150 (5.9%)	64.0%	0.76	0.66	0.88	0.001
	6 to 12 months	116 (4.6%)	57.8%	0.73	0.61	0.87	0.003
	12 to 18 months	76 (3.0%)	47.4%	0.62	0.49	0.79	0.001
	18 to 24 months	138 (5.4%)	19.6%	0.30	0.21	0.44	7E-09
	24 months or more	191 (7.5%)	14.1%	0.19	0.12	0.29	4E-12
	**Symptom duration (prospective logging)**						

	Unknown	335 (13.1%)	62.1%	0.82	0.75	0.90	5E-04
	Asymptomatic	188 (7.4%)	88.8%	1.03	0.95	1.12	0.537
	Less than 2 weeks (reference)	686 (26.9%)	85.6%	1.00			
	2 to 4 weeks	204 (8.0%)	80.9%	0.98	0.90	1.07	0.738
	4 to 12 weeks	576 (22.6%)	70.1%	0.90	0.84	0.97	0.016
	3 to 6 months	232 (9.1%)	46.1%	0.61	0.52	0.72	2E-07
	6 to 12 months	146 (5.7%)	43.2%	0.53	0.42	0.67	2E-06
	12 to 18 months	132 (5.2%)	27.3%	0.38	0.28	0.52	6E-08
	18 to 24 months	32 (1.3%)	28.1%	0.34	0.19	0.60	1E-03
	24 months or more	17 (0.7%)	11.8%	0.13	0.03	0.57	0.020
	**Affected function duration**						
	Able to function as normal	420 (16.5%)	81.4%	0.99	0.93	1.05	0.716
	Less than 2 weeks (reference)	915 (35.9%)	84.4%	1.00			
	2 to 4 weeks	294 (11.5%)	76.2%	0.97	0.89	1.05	0.519
	4 to 12 weeks	248 (9.7%)	69.4%	0.86	0.78	0.95	0.014
	3 to 6 months	170 (6.7%)	60.0%	0.74	0.64	0.86	0.001
	6 to 12 months	105 (4.1%)	58.1%	0.78	0.65	0.94	0.023
	12 to 18 months	59 (2.3%)	50.8%	0.60	0.44	0.83	0.007
	18 to 24 months	147 (5.8%)	12.9%	0.22	0.14	0.35	1E-08
	24 months or more	181 (7.1%)	10.5%	0.17	0.11	0.28	5E-11
	**New conditions due to COVID-19**						
	None (reference)	2279 (89.4%)	74.2%	1.00			
	One	110 (4.3%)	30.9%	0.57	0.42	0.79	3E-03
	Two	66 (2.6%)	22.7%	0.29	0.17	0.48	2E-05
	Three or more	68 (2.7%)	5.9%	0.08	0.03	0.21	9E-06
	**Long COVID Diagnosis**						
	No (no self-reported long COVID)	1446 (56.8%)	90.0%	1.60	1.45	1.75	2E-20
	No (with self-reported long COVID) (reference)	762 (29.9%)	48.8%	1.00			
	Yes	337 (13.2%)	21.4%	0.58	0.45	0.74	1E-04
	**Long COVID care services received**						
	None (reference)	2195 (86.1%)	75.7%	1.00			
	One or more	353 (13.9%)	24.6%	0.73	0.58	0.92	0.020
**Factors unique to cohort**	**First language**						
	English (reference)	2430 (95.4%)	68.7%	1.00			
	Other	47 (1.8%)	78.7%	1.18	0.99	1.41	0.119
	Prefer not to answer/not stated	71 (2.8%)	57.7%	0.87	0.71	1.06	0.268

TwinsUK

Table S 2. **Extended sample characteristics and results of multivariable poisson regression models, TwinsUK cohort.** Relative risk ratios, 95% confidence intervals (CI) and p-values adjusted for multiple testing (Benjamini/Hochberg false discovery rate correction) are presented for multivariable poisson regression models testing association between recovery from COVID-19 and exposure of interest, after adjustment as appropriate from the hypothesised directed acyclic graph (DAG), and weighting for inverse probability of questionnaire participation and selection into analysis sample.

		TwinsUK					
Domain	Variable	Group size, N (%)	COVID-19 recovery (%)	Relative risk ratio	95% CI (lower)	95% CI (upper)	P-value (adjusted)
**Individual pre-pandemic demographics**	**Age group (years)**						
	18-39	266 (19.9%)	88.3%	1.10	0.99	1.21	0.321
	40-49	195 (14.6%)	79.0%	1.02	0.90	1.15	0.923
	50-59 (reference)	338 (25.3%)	78.1%	1.00			
	60-69	294 (22.0%)	79.3%	1.05	0.95	1.15	0.721
	≥ 70	241 (18.1%)	79.7%	1.04	0.95	1.15	0.750
	**Sex**						
	Female (reference)	1153 (86.4%)	79.7%	1.00			
	Male	181 (13.6%)	87.8%	1.11	1.02	1.20	0.093
	**Ethnic group**						
	Asian/Asian British	15 (1.1%)	73.3%	0.86	0.55	1.35	0.831
	Black/Black British	14 (1.0%)	71.4%	1.02	0.78	1.32	0.946
	Mixed/Multiple	17 (1.3%)	82.4%	0.82	0.53	1.27	0.721
	Other	6 (0.4%)	50.0%	0.39	0.12	1.33	0.435
	White (reference)	1282 (96.1%)	81.1%	1.00			
	**Highest educational qualification**						
	Unknown	53 (4.0%)	66.0%	0.77	0.63	0.94	0.093
	Did not complete secondary school	60 (4.5%)	83.3%	1.02	0.87	1.18	0.945
	GCSE or GNVQ or equivalent	269 (20.2%)	78.4%	0.99	0.91	1.09	0.946
	A-Levels or advanced GNVQ or equivalent	351 (26.3%)	77.5%	0.92	0.84	1.00	0.252
	University degree (reference)	386 (28.9%)	84.5%	1.00			
	Postgraduate degree or higher	215 (16.1%)	85.6%	1.00	0.91	1.09	0.965
	**UK Region**						
	East Midlands	69 (5.2%)	72.5%	0.80	0.64	1.00	0.228
	East of England	174 (13.0%)	83.9%	1.03	0.92	1.15	0.890
	London (reference)	266 (19.9%)	83.1%	1.00			
	North East	32 (2.4%)	78.1%	0.91	0.73	1.15	0.780
	North West	93 (7.0%)	86.0%	1.04	0.92	1.18	0.831
	Scotland & Northern Ireland	45 (3.4%)	73.3%	0.83	0.65	1.05	0.427
	South East	310 (23.2%)	80.0%	0.98	0.89	1.07	0.890
	South West	142 (10.6%)	81.0%	0.97	0.85	1.10	0.890
	Wales	47 (3.5%)	70.2%	0.82	0.63	1.06	0.435

	West Midlands	79 (5.9%)	82.3%	0.99	0.87	1.13	0.946
	Yorkshire and The Humber	77 (5.8%)	80.5%	1.00	0.87	1.16	0.990
	**Rural-Urban classification**						
	Rural	289 (21.7%)	78.9%	0.98	0.90	1.07	0.890
	Urban (reference)	1045 (78.3%)	81.3%	1.00			
	**Pre-pandemic employment status**						
	Employed (reference)	689 (51.6%)	82.3%	1.00			
	Self-employed	119 (8.9%)	80.7%	0.98	0.87	1.11	0.932
	Unemployed	9 (0.7%)	100.0%				
	Permanently or long-term sick or disabled	18 (1.3%)	44.4%	0.49	0.27	0.88	0.127
	Retired	279 (20.9%)	80.3%	0.98	0.88	1.09	0.911
	Other	124 (9.3%)	77.4%	0.95	0.84	1.08	0.771
	Unknown	96 (7.2%)	81.2%	1.04	0.94	1.17	0.780
	**Local area deprivation**						
	IMD Decile 1 (most deprived 10%)	45 (3.4%)	75.6%	0.95	0.77	1.18	0.890
	IMD Decile 2	68 (5.1%)	77.9%	0.95	0.80	1.11	0.831
	IMD Decile 3	65 (4.9%)	81.5%	0.99	0.84	1.16	0.946
	IMD Decile 4	102 (7.6%)	81.4%	0.97	0.84	1.11	0.890
	IMD Decile 5	133 (10.0%)	77.4%	0.88	0.76	1.01	0.321
	IMD Decile 6	143 (10.7%)	83.2%	0.98	0.87	1.10	0.923
	IMD Decile 7	155 (11.6%)	81.9%	0.95	0.84	1.07	0.725
	IMD Decile 8	188 (14.1%)	79.8%	0.98	0.88	1.09	0.914
	IMD Decile 9	208 (15.6%)	81.2%	0.99	0.89	1.10	0.932
	IMD Decile 10 (least deprived 10%) (reference)	227 (17.0%)	82.4%	1.00			
**Socio-demographics during pandemic**	**Pandemic adverse experiences**						
	Absence of given experience (reference)			1.00			
	Lost job/unable to work	206 (15.4%)	76.7%	0.91	0.81	1.01	0.330
	Put on furlough (paid leave at ~80% salary)	208 (15.6%)	83.7%	0.98	0.90	1.07	0.890
	Unable to pay bills	78 (5.8%)	71.8%	0.87	0.72	1.05	0.435
	Evicted/lost accommodation	14 (1.0%)	92.9%	1.14	0.97	1.35	0.390
	Unable to access sufficient food	63 (4.7%)	61.9%	0.77	0.62	0.96	0.135
	Unable to access required medication	97 (7.3%)	70.1%	0.85	0.73	1.00	0.228
	Unable to access health services in the community	490 (36.7%)	77.8%	0.96	0.90	1.03	0.658
	Unable to access the community social care services or voluntary sector support needed	38 (2.8%)	60.5%	0.70	0.52	0.95	0.151
	Unable to access inpatient or outpatient appointments booked at a hospital	222 (16.6%)	69.8%	0.88	0.79	0.98	0.127
	Unable to access appointment for cognitive behaviour therapy, counselling, or psychological therapy	70 (5.2%)	68.6%	0.75	0.60	0.93	0.089

	Lost somebody close due to COVID-19	170 (12.7%)	72.9%	0.89	0.79	1.00	0.228
	Lost twin due to COVID-19	5 (0.4%)	60.0%	0.72	0.26	2.02	0.857
	Change in relationship status	63 (4.7%)	81.0%	0.92	0.79	1.06	0.622
	**Number of pandemic adverse health care experiences (of 5)**						
	None (reference)	759 (56.9%)	83.8%	1.00			
	One	328 (24.6%)	82.3%	1.01	0.94	1.08	0.932
	Two	162 (12.1%)	71.0%	0.91	0.81	1.02	0.372
	Three or more	80 (6.0%)	67.5%	0.78	0.64	0.94	0.089
	Unknown	5 (0.4%)	60.0%	0.75	0.32	1.74	0.831
	**Overall number of pandemic adverse experiences (of 13)**						
	None (reference)	518 (38.8%)	84.4%	1.00			
	One	367 (27.5%)	82.8%	0.98	0.91	1.05	0.857
	Two	212 (15.9%)	77.8%	0.93	0.84	1.02	0.371
	Three	112 (8.4%)	83.0%	0.97	0.86	1.10	0.890
	Four or more	121 (9.1%)	62.8%	0.69	0.58	0.82	8E-04
	Unknown	< 5 (0.3%)	75.0%	1.00	0.49	2.03	0.999
	**Current employment status**						
	Employed (reference)	698 (52.3%)	83.2%	1.00			
	Self-employed	109 (8.2%)	81.7%	1.05	0.91	1.21	0.831
	Unemployed	11 (0.8%)	72.7%	0.89	0.61	1.30	0.857
	Permanently or long-term sick or disabled	22 (1.6%)	31.8%	0.45	0.21	0.97	0.215
	Retired	343 (25.7%)	80.2%	1.02	0.89	1.16	0.932
	Other	105 (7.9%)	76.2%	0.99	0.85	1.14	0.946
	Unknown	46 (3.4%)	82.6%	1.10	0.90	1.33	0.721
	**Current household income**						
	Prefer not to answer	140 (10.5%)	77.1%	1.04	0.88	1.23	0.890
	Less than £20,000	123 (9.2%)	74.0%	1.01	0.86	1.18	0.946
	£20,000-£29,999	145 (10.9%)	86.2%	1.16	1.02	1.31	0.163
	£30,000-£39,999	131 (9.8%)	82.4%	1.04	0.91	1.18	0.890
	£40,000-£49,999 (reference)	138 (10.3%)	79.7%	1.00			
	£50,000-£74,999	205 (15.4%)	79.0%	0.95	0.83	1.08	0.750
	£75,000-£99,999	107 (8.0%)	87.9%	1.08	0.94	1.23	0.658
	£100,000 or more	136 (10.2%)	86.0%	1.03	0.91	1.17	0.890
	Unknown	209 (15.7%)	78.0%	1.04	0.91	1.18	0.857
**Pre-pandemic health factors**	**Pre-pandemic health**						
	Poor	17 (1.3%)	35.3%	0.54	0.28	1.04	0.278
	Fair	80 (6.0%)	68.8%	0.93	0.80	1.09	0.721
	Good	295 (22.1%)	77.6%	0.97	0.89	1.05	0.780
	Very good (reference)	458 (34.3%)	82.5%	1.00			
	Excellent	312 (23.4%)	88.1%	1.07	0.99	1.16	0.321
	Unknown	172 (12.9%)	78.5%	0.94	0.80	1.11	0.828
	**Body mass index**						

	< 18.5 kg/m^2	35 (2.6%)	94.3%	1.16	1.06	1.28	0.023
	18.5-25 kg/m^2 (reference)	580 (43.5%)	83.1%	1.00			
	25-30 kg/m^2	359 (26.9%)	80.2%	1.02	0.94	1.10	0.890
	≥ 30 kg/m^2	192 (14.4%)	76.0%	1.00	0.90	1.11	0.999
	Unknown	168 (12.6%)	76.8%	0.96	0.83	1.10	0.857
	**PRISMA-7 Frailty score**						
	0-2, below threshold (reference)	802 (60.1%)	82.0%	1.00			
	3-7, above threshold	43 (3.2%)	67.4%	0.91	0.75	1.10	0.701
	Unknown	489 (36.7%)	80.0%	1.19	0.93	1.52	0.471
	**Number of physical health conditions**						
	None (reference)	703 (52.7%)	82.2%	1.00			
	One	304 (22.8%)	84.2%	1.05	0.98	1.12	0.459
	Two or more	90 (6.7%)	66.7%	0.84	0.71	0.99	0.209
	Unknown	237 (17.8%)	77.6%	1.02	0.92	1.14	0.890
	**Number of mental health conditions (pre-pandemic)**						
	None (reference)	804 (60.3%)	83.8%	1.00			
	One	185 (13.9%)	77.3%	0.91	0.82	1.00	0.215
	Two or more	108 (8.1%)	71.3%	0.81	0.70	0.95	0.089
	Unknown	237 (17.8%)	77.6%	0.99	0.89	1.10	0.945
**COVID-19 illness characteristics**	**Infection period**						
	Before 2020-12-08 (pre-vaccination, wild-type dominant) (reference)	677 (50.7%)	78.0%	1.00			
	2020-12-08 to 2021-04-25 (alpha-variant dominant)	148 (11.1%)	77.7%	1.03	0.92	1.16	0.890
	2021-04-25 to 2021-12-08 (delta-variant dominant)	205 (15.4%)	77.1%	0.99	0.89	1.09	0.940
	After 2021-12-08 (omicron-variant dominant)	304 (22.8%)	91.1%	1.18	1.10	1.26	0.000
	**Hospitalised during COVID-19 illness**						
	No (reference)	1293 (96.9%)	81.9%	1.00			
	Yes	37 (2.8%)	51.4%	0.86	0.64	1.15	0.661
	**Symptom duration (retrospective self-report)**						
	Less than 2 weeks (including asymptomatic) (reference)	603 (45.2%)	93.4%	1.00			
	2 to 4 weeks	289 (21.7%)	85.5%	0.89	0.83	0.95	5E-03
	4 to 12 weeks	217 (16.3%)	76.5%	0.82	0.75	0.89	3E-04
	12 or more weeks	92 (6.9%)	56.5%	0.54	0.42	0.68	2E-05
	3 to 12 months	69 (5.2%)	53.6%	0.55	0.42	0.72	3E-04
	12 or more months	64 (4.8%)	20.3%	0.16	0.09	0.29	7E-08
	**Affected function duration**						
	Unknown	292 (21.9%)	88.0%	0.99	0.89	1.11	0.946
	N/A - No symptoms	77 (5.8%)	85.7%	0.99	0.86	1.12	0.941
	Able to function as normal	224 (16.8%)	86.6%	1.04	0.96	1.13	0.661
	Less than 2 weeks (reference)	430 (32.2%)	85.6%	1.00			
	2 to 4 weeks	122 (9.1%)	72.1%	0.92	0.81	1.05	0.574

	4 to 12 weeks	80 (6.0%)	67.5%	0.84	0.69	1.01	0.272
	12 or more weeks	79 (5.9%)	34.2%	0.44	0.30	0.63	3E-04
	**Long COVID Diagnosis**						
	No (self-reported long COVID status unknown)	76 (5.7%)	68.4%	1.29	1.03	1.63	0.166
	No (no self-reported long COVID)	796 (59.7%)	92.8%	1.63	1.39	1.92	4E-07
	No (with self-reported long COVID) (reference)	184 (13.8%)	45.7%	1.00			
	Yes	47 (3.5%)	23.4%	0.70	0.38	1.27	0.604
**Factors unique to cohort**	**Pre-pandemic household income**						
	Prefer not to answer	143 (10.7%)	73.4%	0.89	0.77	1.04	0.435
	Less than £20,000	102 (7.6%)	82.4%	1.04	0.91	1.20	0.857
	£20,000-£29,999	114 (8.5%)	82.5%	1.03	0.90	1.18	0.890
	£30,000-£39,999	106 (7.9%)	82.1%	1.02	0.89	1.17	0.923
	£40,000-£49,999 (reference)	115 (8.6%)	82.6%	1.00			
	£50,000-£74,999	185 (13.9%)	85.4%	1.02	0.90	1.15	0.932
	£75,000-£99,999	98 (7.3%)	85.7%	0.97	0.83	1.14	0.923
	£100,000 or more	112 (8.4%)	83.0%	0.97	0.84	1.12	0.892
	Unknown	359 (26.9%)	77.4%	0.91	0.81	1.04	0.457
	**Credit/benefit claims before pandemic**						
	None (reference)	1072 (80.4%)	82.6%	1.00			
	One or more	91 (6.8%)	68.1%	0.82	0.69	0.97	0.151
	**Housing tenure**						
	Owned outright	387 (29.0%)	79.6%	1.00			
	Owned with mortgage	302 (22.6%)	80.8%	0.99	0.90	1.09	0.946
	Rented	102 (7.6%)	84.3%	1.05	0.93	1.19	0.761
	Other	55 (4.1%)	90.9%	1.03	0.89	1.21	0.890
	**Housing, trouble with vermin, damp or mould**						
	No (reference)	748 (56.1%)	82.9%	1.00			
	Yes	98 (7.3%)	69.4%	0.82	0.70	0.96	0.098
	**Number of caring responsibilities (of 8)**						
	None (reference)	449 (33.7%)	82.4%	1.00			
	One	335 (25.1%)	81.5%	1.02	0.94	1.10	0.890
	Two	258 (19.3%)	79.5%	1.01	0.92	1.11	0.941
	Three	69 (5.2%)	79.7%	0.98	0.84	1.14	0.932
	Four or more	84 (6.3%)	77.4%	0.99	0.86	1.14	0.946
	**Number of significant stressors (of 16, April-August 2020)**						
	None (reference)	530 (39.7%)	84.0%	1.00			
	One	151 (11.3%)	80.1%	0.92	0.83	1.03	0.435
	Two	165 (12.4%)	80.0%	1.00	0.91	1.10	0.999
	Three or more	308 (23.1%)	76.6%	0.96	0.88	1.05	0.721
	**Credit/benefit claims during pandemic**						
	None (reference)	1080 (81.0%)	82.6%	1.00			

	One or more	209 (15.7%)	74.2%	0.95	0.86	1.05	0.721

MAIHDA intersectional mixed-effects model results tables (corresponding to main text Figure 3)

Stratum-level predicted probabilities

Table S 3. **Stratum-level predicted probabilities of COVID-19 recovery from MAIHDA models.** Unadjusted proportion and predicted probability (with approximate 95% confidence intervals) of self-reported recovery from COVID-19 from MAIHDA mixed-effects logistic regression for combinations of sex, education level and local area deprivation. 95% confidence intervals are approximate only because the model assumes no sampling covariability between the regression coefficients and stratum random effects. Models included participant weighting for inverse probability of questionnaire participation and selection into analysis samples. Strata labels: F = Female, M = Male; NoDegree = Less than degree level education (including not stated/prefer not to say), Degree = Undergraduate degree level or higher; IMDQ1/2/3/4/5 = Index of Multiple Deprivation (IMD) Quintile 1/2/3/4/5, where 1 is most deprived 20% of areas, and 5 is least deprived 20%.

Cohort	Sample	Stratum	Stratum size, N	Unadjusted COVID-19 recovery (%)	Predicted probability of COVID-19 recovery (%)	Lower 95% CI	Upper 95% CI
**CSSB**	**All COVID-19**	F + Degree + IMDQ1	70	67.1	67.7	56.1	76.9
		F + Degree + IMDQ2	185	67.6	69.0	59.8	76.8
		F + Degree + IMDQ3	301	65.8	70.5	61.8	77.8
		F + Degree + IMDQ4	366	74.3	74.4	67.1	80.3
		F + Degree + IMDQ5	489	69.5	73.5	66.4	79.5
		F + NoDegree + IMDQ1	57	54.4	55.1	44.0	65.1
		F + NoDegree + IMDQ2	96	53.1	55.2	44.9	65.3
		F + NoDegree + IMDQ3	126	59.5	62.2	53.7	70.1
		F + NoDegree + IMDQ4	174	66.1	62.6	53.4	71.0
		F + NoDegree + IMDQ5	213	70.9	67.8	59.2	75.3
		M + Degree + IMDQ1	15	73.3	72.8	61.6	81.5
		M + Degree + IMDQ2	32	81.3	73.7	63.2	81.7
		M + Degree + IMDQ3	66	77.3	77.1	68.6	83.7
		M + Degree + IMDQ4	86	75.6	77.7	70.1	83.9
		M + Degree + IMDQ5	135	74.8	79.1	71.8	85.1
		M + NoDegree + IMDQ1	14	78.6	64.4	52.3	75.1
		M + NoDegree + IMDQ2	17	47.1	65.5	52.0	75.5
		M + NoDegree + IMDQ3	30	70.0	68.8	58.7	77.3
		M + NoDegree + IMDQ4	28	60.7	69.9	59.3	78.2
		M + NoDegree + IMDQ5	48	66.7	70.5	60.9	78.2
	**Long COVID**	F + Degree + IMDQ1	36	36.1	38.9	26.7	53.4
		F + Degree + IMDQ2	82	32.9	38.0	27.1	49.8
		F + Degree + IMDQ3	130	37.7	40.3	30.5	51.2
		F + Degree + IMDQ4	149	48.3	49.8	39.9	59.9
		F + Degree + IMDQ5	193	43.0	48.2	38.6	58.2
		F + NoDegree + IMDQ1	32	28.1	30.9	21.4	43.8

		F + NoDegree + IMDQ2	60	28.3	29.7	19.8	41.8
		F + NoDegree + IMDQ3	59	30.5	33.9	25.7	43.4
		F + NoDegree + IMDQ4	85	44.7	40.0	30.1	51.1
		F + NoDegree + IMDQ5	85	40.0	40.3	30.6	50.7
		M + Degree + IMDQ1	<10	50.0	43.0	29.6	58.3
		M + Degree + IMDQ2	14	57.1	43.6	29.4	59.5
		M + Degree + IMDQ3	28	50.0	45.6	34.0	59.2
		M + Degree + IMDQ4	29	34.5	53.6	40.8	66.8
		M + Degree + IMDQ5	49	51.0	53.4	41.8	65.0
		M + NoDegree + IMDQ1	<10	66.7	35.5	23.1	51.0
		M + NoDegree + IMDQ2	<10	0.0	35.1	23.2	49.4
		M + NoDegree + IMDQ3	14	35.7	39.3	28.5	51.6
		M + NoDegree + IMDQ4	16	31.3	47.6	34.5	60.9
		M + NoDegree + IMDQ5	21	33.3	46.2	34.5	58.9
**TwinsUK**	**All COVID-19**	F + Degree + IMDQ1	39	84.6	81.3	67.7	89.8
		F + Degree + IMDQ2	70	91.4	81.0	68.3	89.0
		F + Degree + IMDQ3	104	83.7	78.4	67.1	86.8
		F + Degree + IMDQ4	125	82.4	81.0	71.4	88.4
		F + Degree + IMDQ5	170	82.9	82.7	73.4	89.3
		F + NoDegree + IMDQ1	58	70.7	73.9	61.1	83.0
		F + NoDegree + IMDQ2	74	67.6	75.8	63.4	84.3
		F + NoDegree + IMDQ3	137	78.1	73.3	63.6	81.0
		F + NoDegree + IMDQ4	174	78.2	76.1	68.1	82.8
		F + NoDegree + IMDQ5	202	77.7	78.8	71.4	84.7
		M + Degree + IMDQ1	<10	80.0	86.4	75.6	93.0
		M + Degree + IMDQ2	12	100.0	88.9	77.4	94.7
		M + Degree + IMDQ3	18	94.4	86.5	77.3	92.6
		M + Degree + IMDQ4	24	79.2	86.6	77.8	93.3
		M + Degree + IMDQ5	34	88.2	89.7	82.5	94.1
		M + NoDegree + IMDQ1	11	81.8	82.7	71.7	90.2
		M + NoDegree + IMDQ2	11	90.9	84.4	73.6	91.3
		M + NoDegree + IMDQ3	17	64.7	82.7	71.1	90.2
		M + NoDegree + IMDQ4	20	95.0	84.2	75.3	90.6
		M + NoDegree + IMDQ5	29	96.6	86.6	78.3	92.0

MAIHDA model parameter estimates

Table S 4. **MAIHDA model parameter estimates**. Summary of fixed and random effects parameter estimates for MAIHDA mixed-effects logistic regression models estimating associations between self-reported recovery from COVID-19 and combinations of sex, education level and local area deprivation. Individual level variance set equal to the variance of the standard logistic distribution ($\pi^2/3$).

	**CSSB, all COVID-19**			**CSSB, long COVID**			**TwinsUK, all COVID-19**		
	**Odds Ratio**	**95% CI**	**p**	**Odds Ratio**	**95% CI**	**p**	**Odds Ratio**	**95% CI**	**p**
**Fixed effects: Regression coefficients**									
(Intercept)	2.69	2.05 – 3.54	<0.001	1.05	0.75 – 1.49	0.766	4.17	2.59 – 6.70	<0.001
Age group [18-39]	1.29	0.91 – 1.82	0.147	1.01	0.61 – 1.66	0.973	2.03	1.24 – 3.34	0.005
Age group [40-49]	1.03	0.80 – 1.32	0.813	0.88	0.60 – 1.29	0.525	1.24	0.73 – 2.10	0.431
Age group [50-59] (Reference)	1			1			1		
Age group [60-69]	1.21	0.97 – 1.50	0.084	1.08	0.79 – 1.48	0.624	1.31	0.85 – 2.01	0.227
Age group [≥70]	1.55	1.12 – 2.13	0.007	1.09	0.71 – 1.69	0.69	1.22	0.81 – 1.84	0.346
Sex [Female] (Reference)	1			1			1		
Sex [Male]	1.26	0.97 – 1.63	0.082	1.23	0.88 – 1.73	0.228	1.72	1.12 – 2.66	0.014
Ethnic group [Asian/Asian British groups]	0.48	0.19 – 1.22	0.121	0.17	0.02 – 1.65	0.126	0.41	0.12 – 1.34	0.14
Ethnic group [Black/Black British groups]	1.99	0.27 – 14.62	0.497	2.95	0.36 – 24.02	0.312	2.75	0.47 – 16.10	0.263
Ethnic group [Mixed/multiple ethnic groups]	0.55	0.27 – 1.14	0.108	0.99	0.42 – 2.29	0.974	0.65	0.22 – 1.88	0.421
Ethnic group [Other ethnic group]	0.57	0.29 – 1.12	0.1	0.55	0.21 – 1.46	0.233	0.09	0.01 – 0.51	0.007
Ethnic group [White groups] (Reference)	1			1			1		
Education level [Prefer not to answer/not stated]	0.52	0.37 – 0.75	<0.001	0.49	0.29 – 0.82	0.006	0.34	0.20 – 0.56	<0.001
Education level [Less than University degree or equivalent]	0.64	0.50 – 0.83	0.001	0.66	0.47 – 0.91	0.012	0.79	0.54 – 1.15	0.218
Education level [Undergraduate degree level] (Reference)	1			1			1		
Education level [Postgraduate degree or higher]	1.00	0.80 – 1.24	0.975	0.8	0.58 – 1.09	0.154	0.83	0.48 – 1.42	0.488
IMD Quintile [1]	0.69	0.47 – 1.02	0.066	0.67	0.40 – 1.12	0.128	0.72	0.43 – 1.21	0.213
IMD Quintile [2]	0.71	0.51 – 0.99	0.042	0.65	0.43 – 0.99	0.046	0.84	0.52 – 1.35	0.461
IMD Quintile [3]	0.85	0.62 – 1.15	0.279	0.73	0.51 – 1.04	0.077	0.71	0.48 – 1.05	0.088

IMD Quintile [4]	0.91	0.68 – 1.22	0.543	1.04	0.74 – 1.45	0.831	0.85	0.59 – 1.23	0.39
IMD Quintile [5] (Reference)	1			1			1		
**Random effects: Variances**									
Individual level	3.29			3.29			3.29		
Stratum level	0.0109			9.31E-06			3.53E-08		
**Summary statistics**									
Variance partition coefficient	0.00330			2.83E-06			1.07E-08		
N, strata	20			20			20		
N, individuals	2548			1105			1334		

Work and social adjustment scale (WSAS) by COVID-19 recovery and long COVID diagnosis status

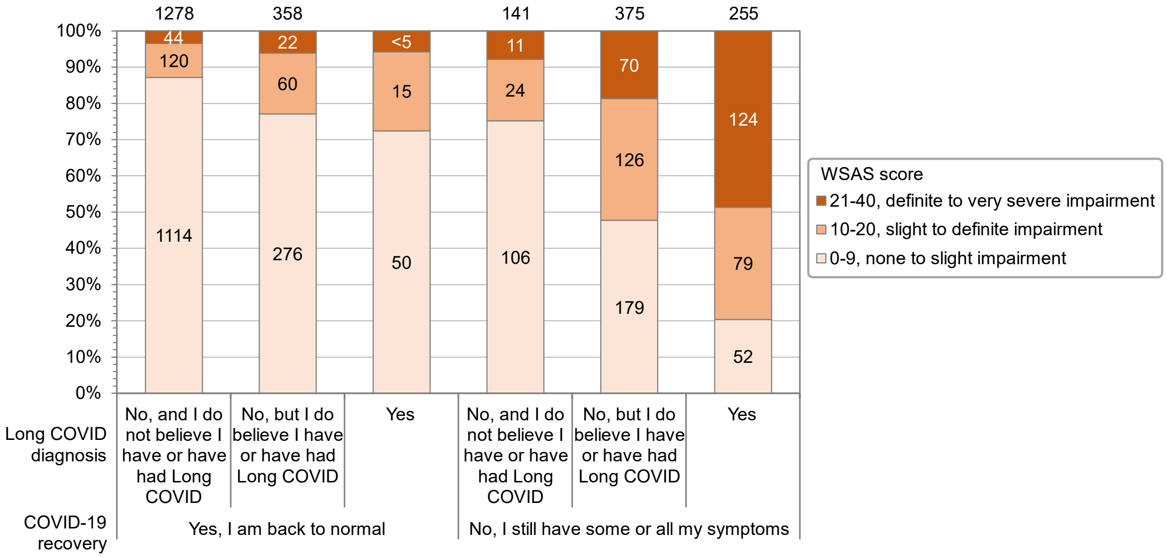

Figure S 4. **Work and social adjustment scale (WSAS) by COVID-19 recovery and long COVID diagnosis status among CSSB participants.** Results shown for all CSSB participants with self-reported COVID-19 infection who completed the WSAS assessment. Data labels show the sample sizes, including totals above each bar. Sample sizes < 5 and associated totals are suppressed.

The effect of pre-pandemic socio-demographics on recovery under alternative directed acyclic graph scenarios

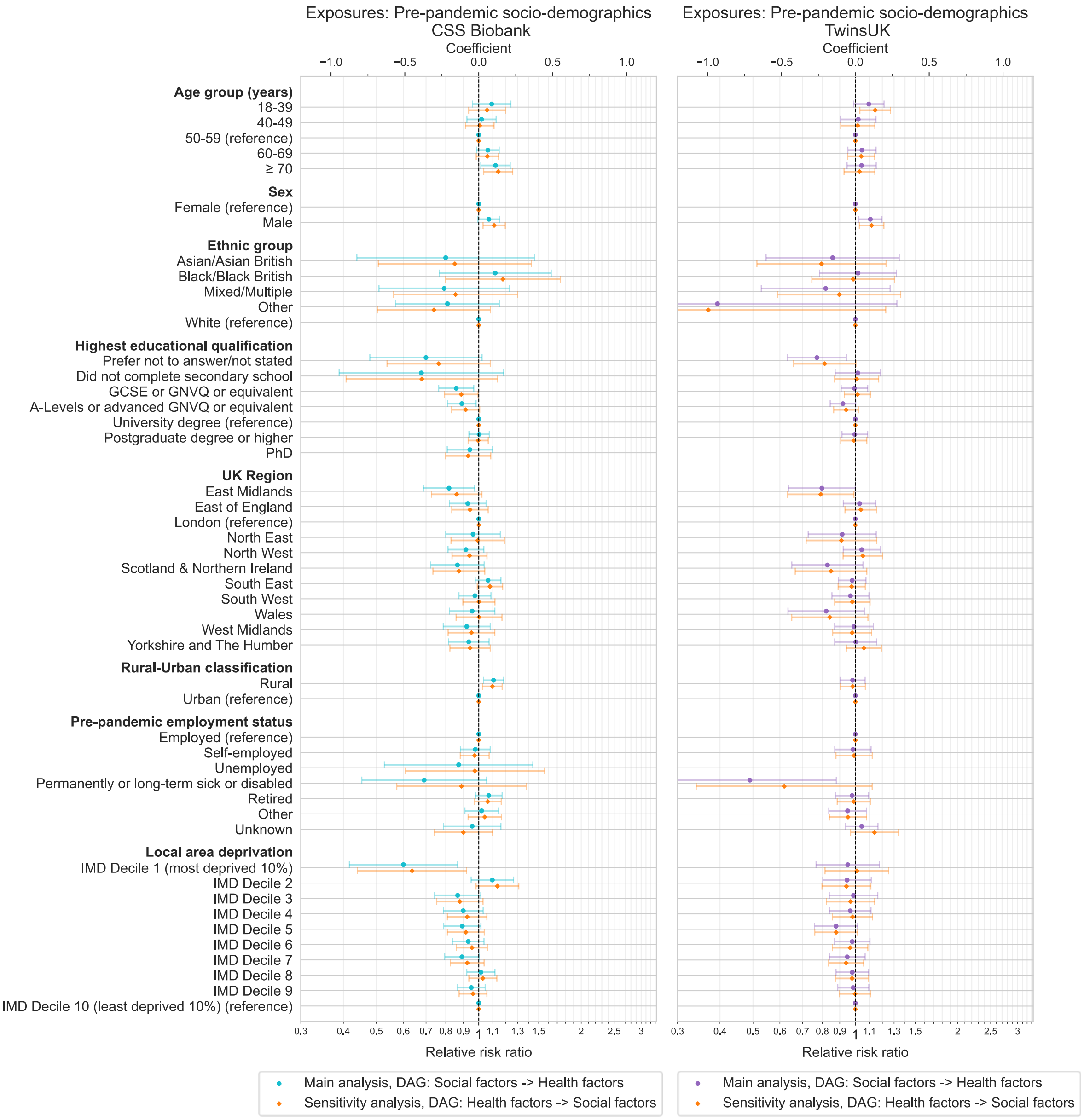

Figure S 5. **Total causal effect estimates for pre-pandemic socio-demographics on recovery from COVID-19 for alternative directed acyclic graph data generation scenarios**. Relative risk ratios and 95% confidence intervals from poisson regression models testing association between recovery from COVID-19 and various pre-pandemic socio-demographic exposure variables, among individuals with self-reported COVID-19 infection. Results are presented for two data generation scenarios, firstly where social factors precede (cause) health factors in time, as presented in the main text, and an alternative where health factors precede (cause) social factors. In the latter models, the following health factors were added to adjustment variable sets: pre-pandemic health, body mass index, number of physical health conditions, PRISMA-7 frailty index, number of mental health conditions [TwinsUK only]. Models included participant weighting for inverse probability of questionnaire response and selection into analysis sample.

Associations between health characteristics and recovery

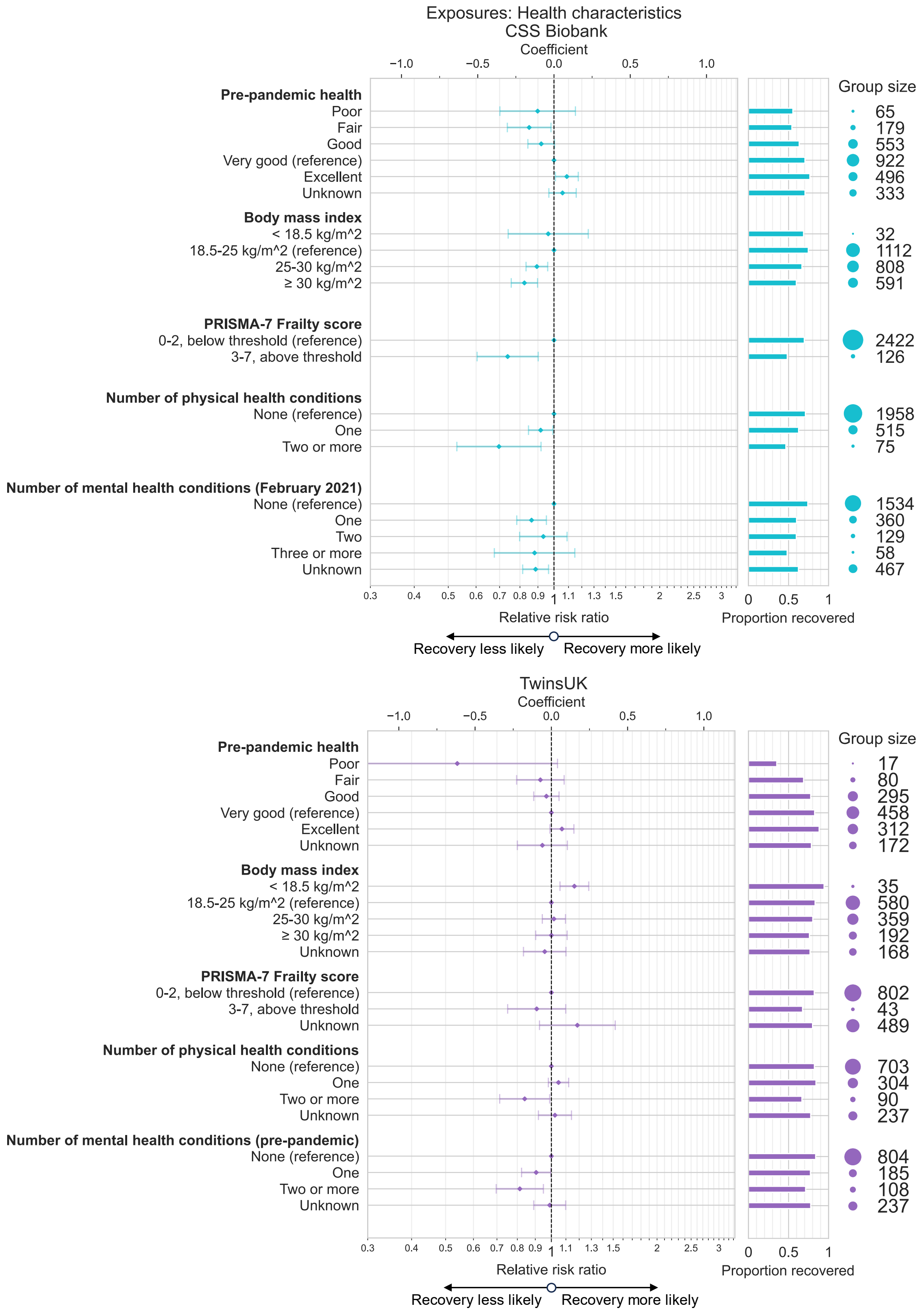

Figure S 6. **Associations between health characteristics and recovery from COVID-19 in CSS Biobank and TwinsUK cohorts**. Relative risk ratio and 95% confidence intervals from poisson regression models testing association between recovery from COVID-19 and various health-related exposure variables, among individuals with self-reported COVID-19 infection. Results for each exposure variable originate from models with distinct adjustment variable sets, including pre-pandemic socio-demographic factors as potential confounding factors as appropriate according to the proposed directed acyclic graph. Models included participant weighting for inverse probability of questionnaire response and selection into analysis sample.

Associations with variables unique to cohort

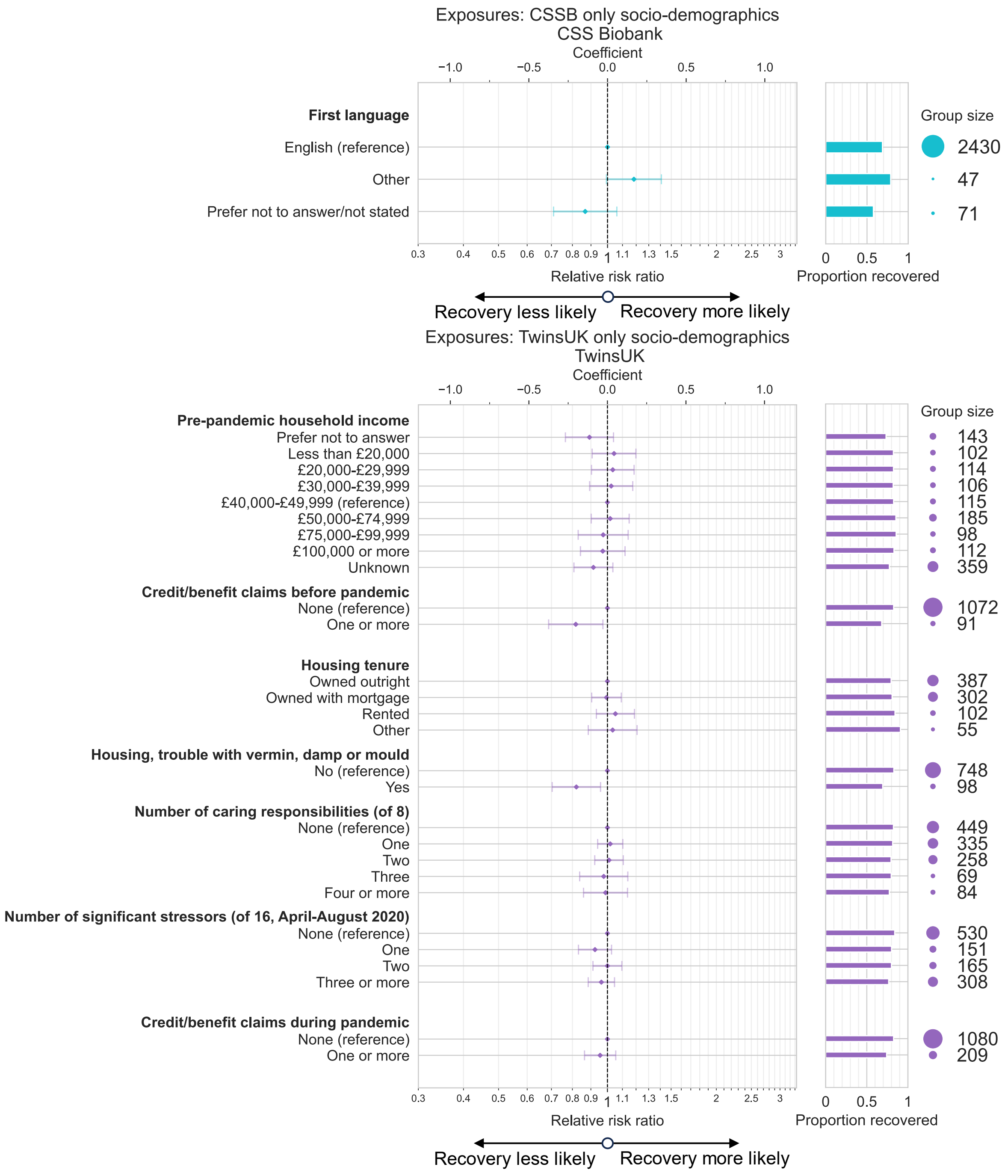

Figure S 7. **Associations between cohort-specific socio-demographics and recovery from COVID-19 in CSS Biobank and TwinsUK cohorts**. Relative risk ratio and 95% confidence intervals from poisson regression models testing association between recovery from COVID-19 and socio-demographic exposure variables unique to each cohort, among individuals with self-reported COVID-19 infection. Results for each exposure variable originate from distinct models, including age, sex, ethnic group, education, pre-pandemic socio-demographic factors and pre-pandemic health characteristics as potential confounding factors.

Association between social strata and COVID-19 recovery adjusting for pre-pandemic health factors

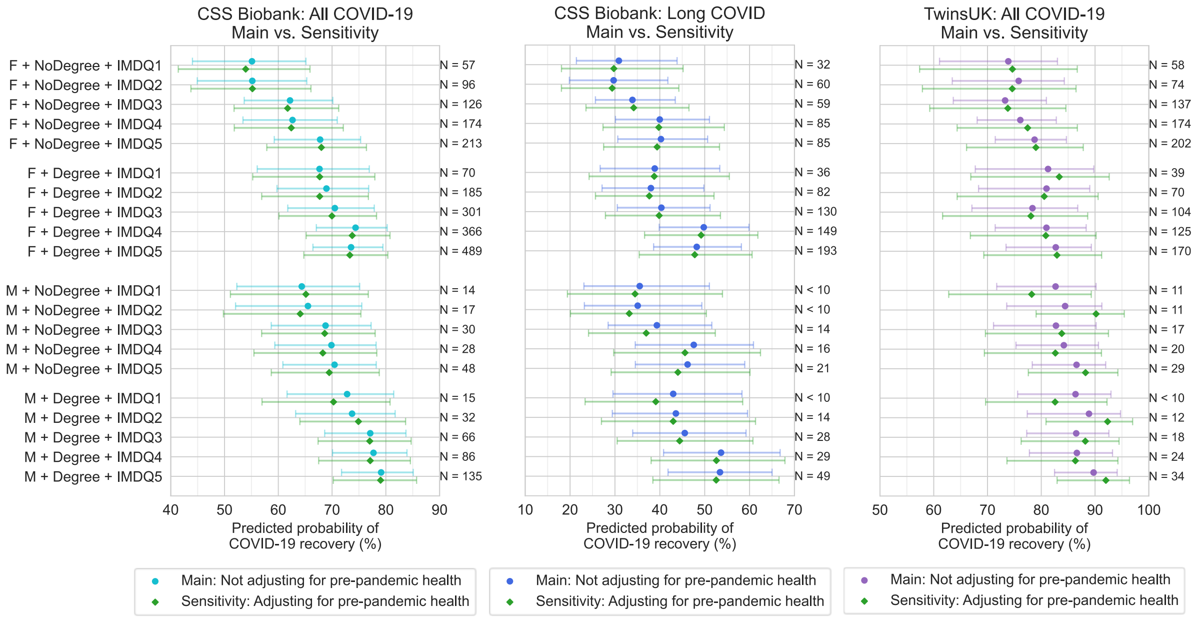

Figure S 8. **Predicted probability of COVID-19 recovery for social strata of sex, education level and local area deprivation from MAIHDA models, including adjustment for pre-pandemic health factors**. Predicted probabilities and approximate 95% confidence intervals from MAIHDA mixed-effects logistic regression models testing association between recovery from COVID-19 and social strata. Results are shown for all from the CSSB cohort participants with self-reported COVID-19 (left), all CSSB participants with diagnosed or self-reported long COVID (centre), and all TwinsUK participants with COVID-19 (right). Results are presented for models that do not adjust for pre-pandemic health factors, as presented in the main text, and models that do adjust (an alternative data generation scenarios where health factors precede (cause) social factors). Fixed-effects included in main analysis models were age group, ethnic group, sex, education level and local area deprivation. In sensitivity models, the following health factors were also included as fixed-effects in MAIHDA models: pre-pandemic health, body mass index, number of physical health conditions, PRISMA-7 frailty index, number of mental health conditions [TwinsUK only]. 95% confidence intervals are approximate only because the model assumes no sampling covariability between the regression coefficients and stratum random effects. Strata labels: F = Female, M = Male; NoDegree = Less than degree level education (including not stated/prefer not to say), Degree = Undergraduate degree level or higher; IMDQ1-IMDQ5 = Index of Multiple Deprivation Quintile 1 to 5, where 1 is most deprived 20% of areas, and 5 is least deprived 20%. Models included participant weighting for inverse probability of questionnaire response and selection into analysis samples.

Associations with COVID-19 illness characteristics

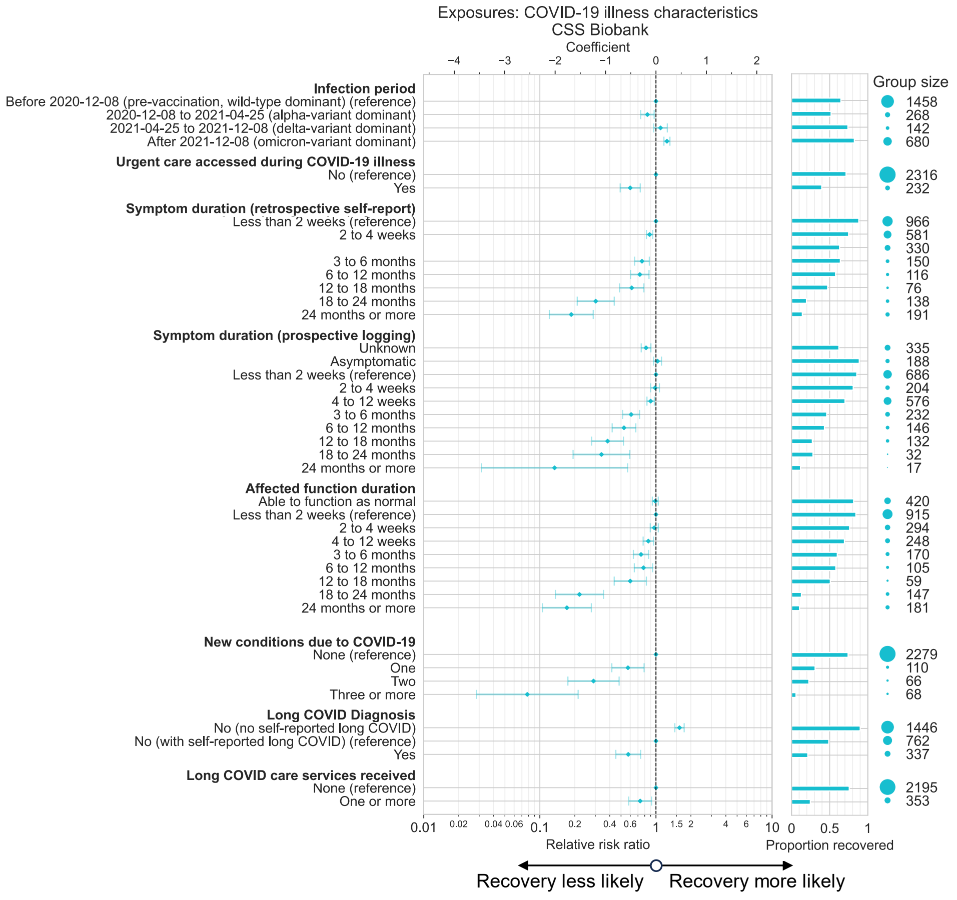

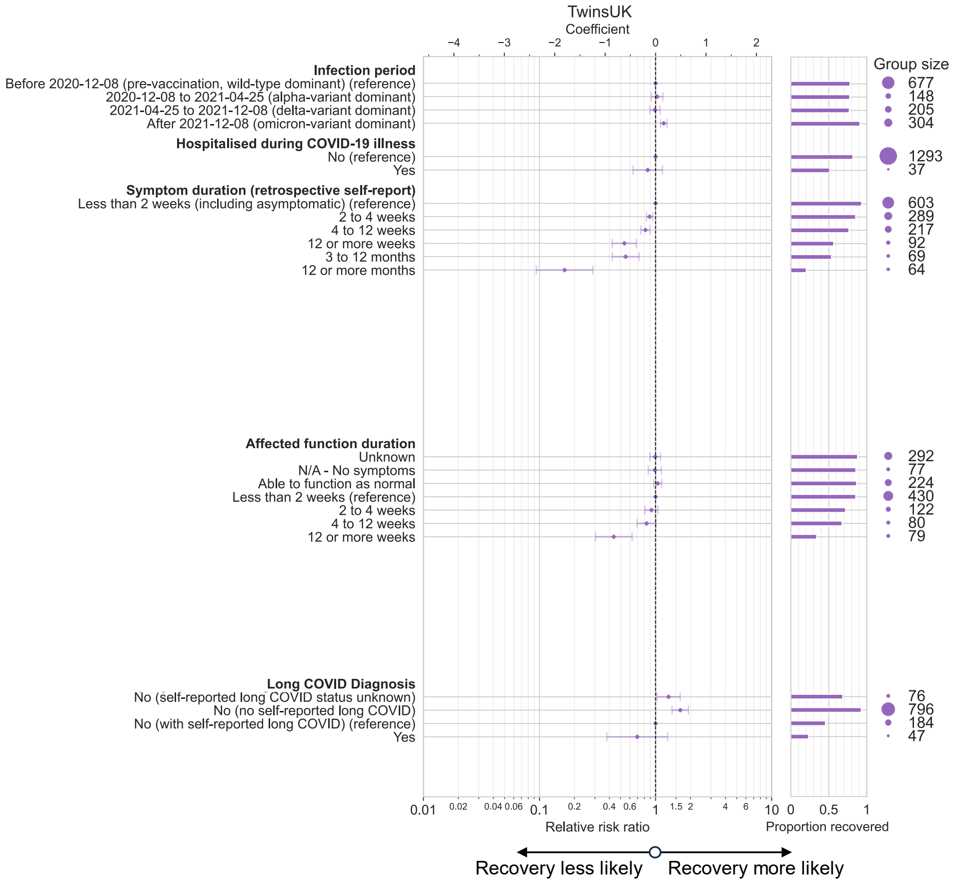

Figure S 9. **Associations between COVID-19 illness characteristics and recovery from COVID-19 in CSS Biobank and TwinsUK cohorts**. Relative risk ratio and 95% confidence intervals from poisson regression models testing association between recovery from COVID-19 and various COVID-19 illness-related exposure variables, among individuals with self-reported COVID-19 infection. Results for each exposure variable originate from models with distinct adjustment variable sets, including pre-pandemic socio-demographic and health factors and socio-demographics collected during the pandemic as potential confounding factors, as appropriate according to the proposed directed acyclic graph. Models included participant weighting for inverse probability of questionnaire response and selection into analysis sample.

References

1 List of ethnic groups - GOV.UK. https://www.ethnicity-facts-figures.service.gov.uk/style-guide/ethnic-groups (accessed 2 Mar 2023).

2 English indices of deprivation 2019 - GOV.UK. https://www.gov.uk/government/statistics/english-indices-of-deprivation-2019 (accessed 29 Nov 2021).

3 Welsh Index of Multiple Deprivation (full Index update with ranks): 2019 | GOV.WALES. https://gov.wales/welsh-index-multiple-deprivation-full-index-update-ranks-2019 (accessed 29 Nov 2021).

4 Scottish Index of Multiple Deprivation 2020 - gov.scot. https://www.gov.scot/collections/scottish-index-of-multiple-deprivation-2020/ (accessed 29 Nov 2021).

5 Northern Ireland Multiple Deprivation Measure 2017 (NIMDM2017) | Northern Ireland Statistics and Research Agency. https://www.nisra.gov.uk/statistics/deprivation/northern-ireland-multiple-deprivation-measure-2017-nimdm2017 (accessed 29 Nov 2021).

6 Xue Q-L. The Frailty Syndrome: Definition and Natural History. *Clinics in Geriatric Medicine* 2011;**27**:1–15. doi:10.1016/j.cger.2010.08.009

7 Raîche M, Hébert R, Dubois MF. PRISMA-7: A case-finding tool to identify older adults with moderate to severe disabilities. *Archives of Gerontology and Geriatrics* 2008;**47**:9–18. doi:10.1016/J.ARCHGER.2007.06.004

8 Mundt JC, Marks IM, Shear MK, *et al.* The Work and Social Adjustment Scale: a simple measure of impairment in functioning. *The British Journal of Psychiatry* 2002;**180**:461–4. doi:10.1192/BJP.180.5.461

9 Cheetham NJ, Penfold R, Giunchiglia V, *et al.* The effects of COVID-19 on cognitive performance in a community-based cohort: a COVID symptom study biobank prospective cohort study. *eClinicalMedicine* 2023;**62**:102086. doi:10.1016/j.eclinm.2023.102086
